# YAP Inhibits HIV-1 transcription and promotes HIV-1 latency by regulating E3 ubiquitin ligase UHRF1 mediated tat degradation

**DOI:** 10.1371/journal.ppat.1013906

**Published:** 2026-01-30

**Authors:** Chenliang Zhou, Hong He, Yuzai Zhang, Xiaolian Liu, Muye Xia, Jiayin Qiu, Ziyao Wu, Huba Khamis Rashid, Wenli Liu, Jie Peng, Lin Li

**Affiliations:** 1 Guangdong Provincial Key Laboratory of New Drug Screening, Key Laboratory of Infectious Diseases Research in South China, Ministry of Education, NMPA Key Laboratory for Research and Evaluation of Drug Metabolism, School of Pharmaceutical Sciences, Southern Medical University, Guangzhou, P. R. China; 2 College of Pharmacy, Nanchang Medical College, Nanchang, P. R. China; 3 The Affiliated Eye Hospital, Jiangxi Medical College, Nanchang University, Nanchang, P. R. China; 4 State Key Laboratory of Organ Failure Research, Guangdong Provincial Key Laboratory of Viral Hepatitis Research, Department of Infectious Diseases, Nanfang Hospital, Southern Medical University, P. R. China; 5 Department of Pharmacy, Zhejiang Chinese Medical University, Hangzhou, Zhejiang, P. R. China; University of Illinois Chicago, UNITED STATES OF AMERICA

## Abstract

The latency of human immunodeficiency virus type 1 (HIV-1) is a major barrier to achieving an HIV-1 cure, as antiretroviral therapy does not target the latent virus. Virus-host interactions play an essential role in various stages of the HIV-1 lifecycle. Exploring the interaction between host factors and HIV-1 infection is critical for developing new HIV-1 treatment strategies. Yes-associated protein (YAP) is a key co-transcription factor in the Hippo signaling pathway, which regulates the occurrence and development of various diseases, including cellular metabolism, cancer, immunity, and viral infection. In this study, we first confirmed that YAP gene expression in patients with acquired immune deficiency syndrome (AIDS) was significantly lower than that in the healthy control group, as determined using the GEO2R online tool. Furthermore, YAP was identified as a negative regulator of HIV-1 transcription by mediating K33- and K48-linked ubiquitination and proteasomal degradation of Tat. Here, we further confirmed that the YAP TAD domain recruited ubiquitin-like with PHD and RING finger domain 1 (UHRF1) to mediate Tat’s ubiquitination and degradation by the screening of the BioGRID database combined with IP-MS analysis. The conserved lysine residues K28, K29, and K41 on Tat were critical acceptor sites for ubiquitination and proteasomal degradation. Our findings revealed that YAP promotes the suppression of HIV-1 transcription and the maintenance of HIV-1 latency, providing novel insights into virus-host interactions for regulating HIV-1 latency.

## Author summary

HIV-1 latency, a dormant state in which the virus remains in cells unnoticed by antiretroviral therapy, is the reason why HIV-1 is still incurable. New HIV-1 therapeutics depend on an understanding of how host factors regulate the virus. Here, we focus on Yes-associated protein (YAP), which controls cellular functions such as viral infection and immunology. We found that YAP inhibits HIV-1 by specifically targeting the viral protein Tat, which is essential for HIV-1 replication. Especially, Tat is destroyed when YAP enhances Tat ubiquitination with the help of host protein UHRF1. The three crucial locations on Tat (K28, K29, and K41) have been certified to be crucial for Tat ubiquitination and subsequent degradation. Our research reveals a new mechanism by which YAP maintains HIV-1 latency and highlights host-virus interactions as promising targets for future HIV-1 cure.

## Introduction

Combined antiretroviral therapy (cART) has been proven to effectively control the progression of acquired immune deficiency syndrome (AIDS), improve patients’ quality of life, and prolong their survival time. Due to the persistence of latent HIV-1 reservoirs, cART therapy cannot completely cure HIV-1 infection, and the discontinuation of cART will invariably lead to a rebound of viremia [1–5]. Numerous literature reports suggest that multiple host factors can mediate HIV-1 transcription and latency [6–8]. Exploring the intricate interactions between host factors and HIV-1 infection is crucial for developing new strategies for HIV-1 treatment.

Yes-associated protein (YAP) is a key effector protein in the Hippo signaling pathway, closely associated with the onset and progression of various diseases [9]. It is worth mentioning that the YAP protein can regulate the transcription and replication of various viruses, including the hepatitis B virus (HBV), the human papillomavirus (HPV), the influenza A virus (IAV), the Kaposi’s sarcoma-associated herpesvirus (KSHV), and so on [10–13]. YAP can reduce the production of antiviral cytokines and promote viral immune escape by affecting the innate immune signaling pathway, thereby regulating viral transcription and replication nonspecifically. The study also revealed that YAP binds to TEAD proteins and cooperatively activates the latent Epstein-Barr virus (EBV) [14]. The Hippo signaling pathway exerts a direct regulatory effect on the replication of Zika virus (ZIKV) and Ebola virus (EBOV) [15,16]. Co-knockdown of YAP/TAZ significantly reduces ZIKV and EBOV replication in host cells. Recent studies also demonstrate that YAP suppresses both human cytomegalovirus (HCMV) and human T-lymphotropic virus type 1 (HTLV-1) infection. YAP inhibits nuclear translocation of HCMV genome by downregulating STING protein levels, while the interaction between YAP and HTLV-1 TAX protein (a HTLV-1 transcriptional activator) disrupts TAX-mediated transcriptional initiation [17–19]. Taken together, those findings indicate that YAP affects the lifecycle of different viruses through divergent regulatory effects, and possibly exhibits strikingly opposite biological effects in its regulatory roles.

In this study, we first identified that the YAP gene expression in patients with AIDS was significantly lower than that in the healthy control group using the GEO2R online tool. Further studies have certified that YAP induces Tat ubiquitination and degradation by recruiting E3 ubiquitin ligase (a ubiquitin-like with PHD and RING finger domain 1 (UHRF1)), thereby inhibiting HIV-1 transcription and promoting HIV-1 latency.

## Results

### YAP inhibits HIV-1 replication and transcription

**Fig 1:**
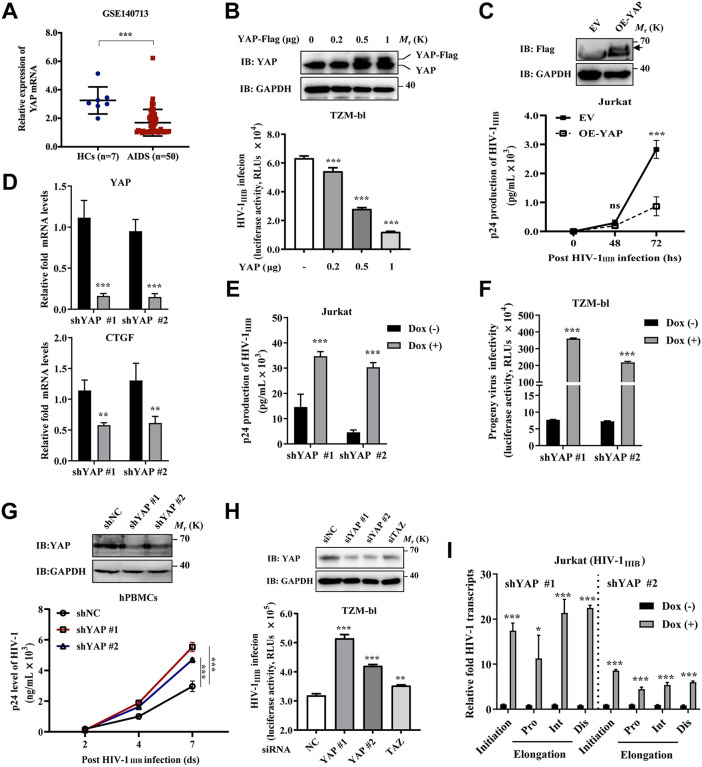
YAP inhibits HIV-1 replication and transcription. **A)** Differential expression of the YAP gene in both AIDS patients and healthy controls (HCs) in the GEO database (GSE140713) was analyzed via the GEO2R online tool. **B)** Immunoblot analysis (top) showing the levels of YAP expression in TZM-bl transfected with the increased amounts of *p*cDNA3.1-YAP-Flag plasmid. HIV-1 LTR-driven luciferase activity in HIV-1_IIIB_-infected TZM-bl cells transfected with the indicated amounts of *p*cDNA3.1-YAP-Flag is shown in the bottom. **C)** Immunoblot analysis (top) showing the levels of Flag-tag expression in Jurkat T cells stably transduced with lentivirus. The band indicated by the arrow is the Flag-tag species. Jurkat T cells stably expressing YAP-Flag or the lentiviral vector were infected with HIV-1_IIIB,_ and the p24 production of the supernatants was detected by ELISA (bottom). **D)** RT-qPCR analysis of the YAP knockdown efficiency in Jurkat T cells generated by a doxycycline (dox)-inducible Tet-On system (CTGF, a classic YAP target gene) following treatment of Jurkat T cells with the HIV-1_IIIB_ virus with or without dox for 48 **h.** **E**, **F)** The p24 production and a luciferase activity were measured by ELISA and a luciferase assay. **G)** Immunoblot analysis (top) showing the levels of YAP expression in hPBMCs isolated from HIV-negative participants transduced with shRNA targeting YAP. The p24 production in the supernatant of HIV-1_IIIB_ infected hPBMCs was shown in the bottom. **H)** Immunoblot analysis (top) showing the levels of YAP expression in TZM-bl transfected with the indicated siRNAs. HIV-1 LTR-driven luciferase activity in HIV-1_IIIB_-infected TZM-bl cells transfected with siRNAs targeting YAP and TAZ was shown in the bottom. **I)** RT-qPCR analysis of the expression of HIV-1 transcripts at different sites (initiation, proximal (Pro), intermediate (Int), and distal (Dis)) of HIV-1_IIIB_ infected Jurkat T cells with or without Dox for 48 **h.** Data were normalized to the values of the control group and were presented as the means ± SDs. To detect significant differences, one-way ANOVA was conducted in panels **A)**, **B)**, and **H)**, and two-way ANOVA was conducted in panels **C)**, **D)**, **E)**, **F)**, **G)**, and **I)** (*P < 0.05, **P < 0.01, ***P < 0.001).

Here, we first analyzed the differential mRNA expression of YAP in the healthy controls (HCs) and AIDS patients in the mRNA microarray dataset (GSE140713) via the GEO2R online tool. Despite the limited sample size, analysis revealed significantly lower YAP gene expression in AIDS patients (n = 50) relative to healthy controls (n = 7) (Fig 1A). However, no significant difference in YAP expression was observed between AIDS patients with low viral load (LVL) and those with high viral load (HVL) (S1 Fig). Nevertheless, we wonder to clarify the impact of YAP on HIV-1 infection. We determined the effects of YAP on the infection by a replication-competent HIV-1_IIIB_ strain in both HeLa-derived TZM-bl cells containing HIV-1 LTR-driven luciferase reporter gene and Jurkat T cells. As shown in Fig 1B, the exogenous YAP-Flag protein was successfully expressed, and the luciferase activity of TZM-bl cells after YAP transfection was significantly decreased in a dose-dependent manner, from approximately 15% to 80%. Furthermore, Jurkat T cells were stably transduced with YAP-Flag or the empty vector (EV), and the YAP overexpression levels were detected by immunoblot analysis (Fig 1C). At 72 hours of HIV-1_IIIB_ infection, the level of p24 production in the supernatants of Jurkat T cells stably overexpressing YAP (OE-YAP) was significantly lower than that in the supernatants of Jurkat T cells transfected with the EV (Fig 1C). We further determined the effect of YAP depletion on HIV-1 infection. As shown in Fig 1D, YAP was successfully depleted in Jurkat T cells using two YAP-specific short hairpin RNAs (shRNAs) (shYAP #1 and #2) lentiviruses by the doxycycline (Dox)-inducible Tet-On system. CTGF, a classical YAP target gene, was used to validate the knockdown of YAP. YAP depletion in the presence of Dox significantly increased the production of p24 in the supernatants of Jurkat T cells (Fig 1E). For testing the infectivity of the progeny viruses, the released virion particles in the supernatants of Jurkat T cells were further added to TZM-bl cells and incubated for another 48 h. As shown in Fig 1F, YAP depletion in the presence of Dox significantly increased the infectivity of progeny viruses in TZM-bl cells. In addition, human PBMCs (hPBMCs) isolated from the blood of healthy donors were stably transduced with two lentiviruses encoding YAP-specific shRNAs. The efficiency of endogenous YAP knockdown in hPBMCs was shown in Fig 1G. YAP knockdown also increased HIV-1_IIIB_ replication in hPBMCs after 7 days postinfection as quantified by ELISA (Fig 1G). Moreover, endogenous YAP was also successfully depleted in TZM-bl cells using two different small YAP-specific siRNAs (siYAP #1 and #2) (Fig 1H). Compared with siNC treatment, the siYAP group modestly increased the luciferase activity, indicating that YAP depletion promotes HIV-1 replication (Fig 1H). The transcriptional co-activator with PDZ-binding motif (TAZ) is an ortholog of YAP, lacking three domains of YAP. Results showed that both YAP and its ortholog TAZ inhibited HIV-1 replication, indicating that YAP might be a limiting factor for HIV-1 replication.

Furthermore, the effects of YAP on the mRNA expression of HIV-1_IIIB_ transcripts at different sites (initiation, proximal (Pro), intermediate (Int), and distal (Dis)) in Jurkat T cells at 48 h postinfection were detected by RT-qPCR analysis. As shown in Fig 1I, YAP knockdown obviously increased the initiation and elongation of HIV-1-LTR-driven transcription.

### YAP promotes HIV-1 latency and suppresses latent HIV-1 reactivation

**Fig 2:**
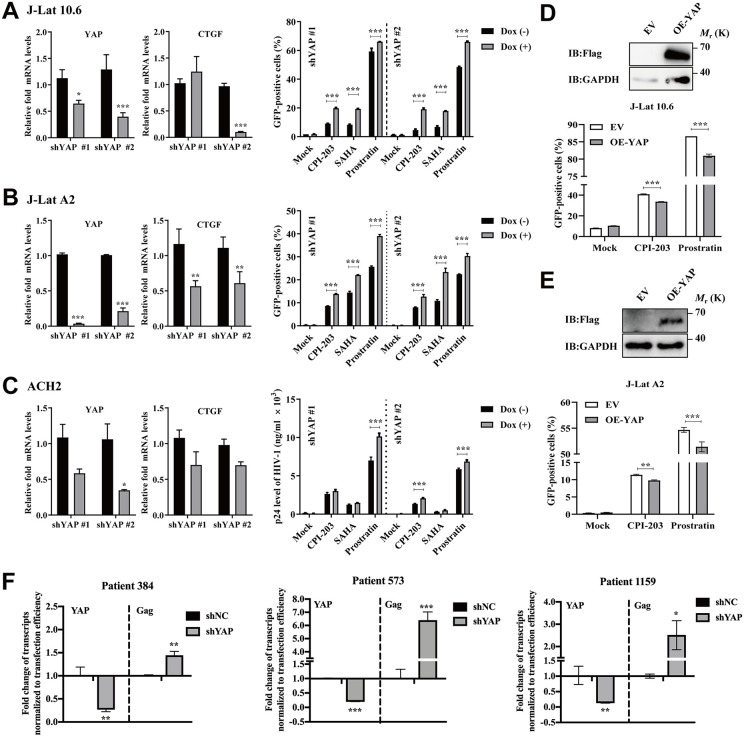
YAP suppresses latent HIV-1 reactivation. **A-C**) HIV-1 latently infected cell lines were infected with the Tet-pLKO-shYAP lentivirus, and the level of YAP expression was determined by RT-qPCR (left). The cells were stimulated with or without LRAs for 48 h, and the GFP^+^ cells **A**) and **B)**, or the level of p24 expression **C)**, were determined by flow cytometry or ELISA (right). **D**, **E)** Immunoblot analysis (top) showing the level of Flag-tag expression in J-Lat 10.6 and J-Lat A2 cells stably expressing Flag-YAP or the lentiviral vector. The cells were stimulated with or without LRAs for 48 h, and the percentage of GFP^+^ cells was summarized (bottom). **F)** RT-qPCR analysis exhibiting the mRNA level of YAP and Gag in CD4^+^ T cells isolated from cART-treated study participants living with HIV-1 (n = 3 donors). Those CD4^+^ T cells were stably transduced with YAP shRNA and stimulated by TNFα (10 ng/mL). Data were normalized to the values of the control group and were presented as the means ± SDs. Two-way ANOVA was conducted to detect significant differences (*P < 0.05, **P < 0.01, ***P < 0.001).

Given the potential negative regulatory role of YAP in HIV-1 transcription and infection, we further investigated its role in the maintenance of HIV-1 latency. Stable latently infected J-Lat T cells (10.6 and A2) and chronically infected ACH2 cells were infected with YAP-specific shRNA lentiviruses with/without the Dox-inducible Tet-On system to knock down endogenous YAP. CTGF was used to validate the knockdown of YAP. The efficiency of endogenous YAP knockdown in the above-described cells was shown in Fig 2A-2C. Here, we chose three typical latency-reversing agents (LRAs) to reactivate the latent HIV-1 on all three tested cells, including a bromodomain and extraterminal domain (BET) inhibitor CPI203, a histone deacetylase (HDAC) inhibitor SAHA, and a protein kinase C (PKC) activator prostratin. Our results revealed that Dox-induced YAP depletion significantly promoted the reactivation of latent HIV-1 on different LRAs-stimulated cells (Fig 2A-2C). Consistently, the effect of YAP knockdown to promote the reactivation was also confirmed in J-Lat A2 cells infected with pLKO.1-shYAP-mCherry lentivirus (S2 Fig). Consistent with these findings, overexpression of YAP in both J-Lat 10.6 (Fig 2D) and J-Lat A2 cells (Fig 2E) conversely decreased the percentage of GFP-positive cells after the treatment of CPI-203 and prostratin. Furthermore, we tested the effect of YAP on HIV-1 latency in primary CD4^+^ T cells isolated from PBMCs of three HIV-1-infected patients on suppressive ART for at least three years with undetectable viral loads (<100 copies/mL) and high CD4^+^ T-cell counts (>500 cells/mL). The CD4^+^ T cells were transfected with lentiviruses encoding YAP-specific shRNA and stimulated with TNF-α for 3 days. Total RNA was extracted and detected by RT-qPCR analysis. The results revealed that depletion of the YAP gene significantly elevated the level of HIV-1 Gag in the culture supernatants in primary CD4^+^ T cells of three patients (Fig 2F). Besides, a rescue experiment demonstrates that the LTR activity is directly attributable to the loss and recovery of YAP function (S3 Fig). These results suggest that YAP plays a key role in maintaining HIV-1 latency, and its depletion benefits latent HIV-1 reactivation stimulated by classical LRAs.

### Serum starvation promoted latent HIV-1 reactivation by regulating YAP

**Fig 3:**
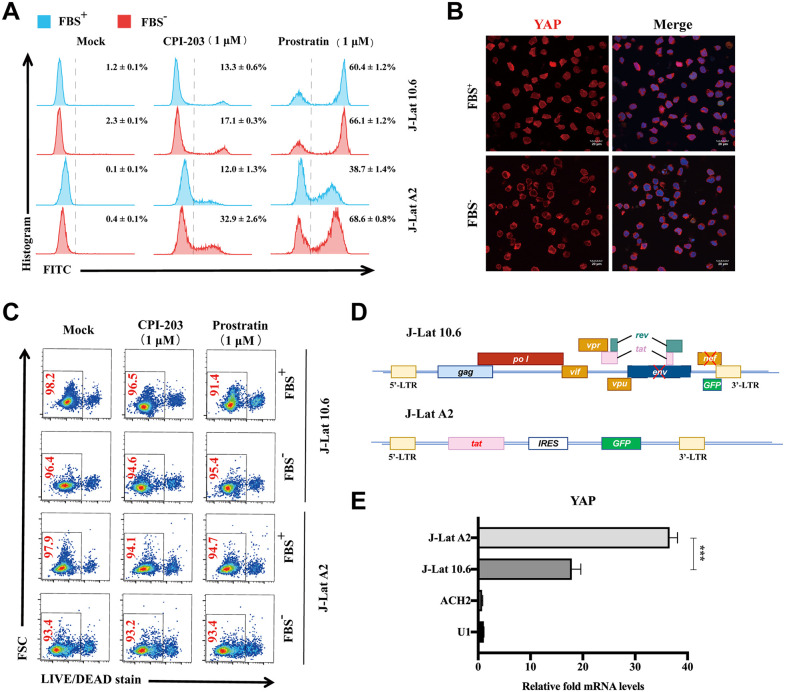
Serum starvation promoted latent HIV-1 reactivation. **A)** The ratio of GFP-positive cells gated in the live cell population following the treatment of J-Lat cell lines with prostratin (1 μM) or CPI-203 (1 μM) in the presence or absence of FBS for 48 **h. B)** Representative images of the expression and location of YAP in J-Lat A2 cells in the presence or absence of FBS. (Scale bar, 20 μm.) **C)** The percentage of cell viability of LRAs-treated J-Lat cell lines in the presence or absence of FBS analyzed by flow cytometry. **D)** Schematic diagram of the HIV-1 minigenome integrated into the J-Lat 10.6 and A2 cell lines. **E)** RT-qPCR analysis of the YAP mRNA expression level in J-Lat A2, J-Lat 10.6, ACH2, and U1. Data were normalized to the values of the control group and are presented as the means ± SDs. To detect significant differences, one-way ANOVA was conducted in panels **E)** (*P < 0.05, **P < 0.01, ***P < 0.001).

Recent studies showed that YAP activation is sufficient to overcome the restriction of global protein synthesis induced by serum starvation [20,21]. To further decipher the possible mechanism(s) by which YAP inhibits HIV-1 replication and promotes HIV-1 latency, we firstly detected the effect of serum starvation on HIV-1 latency. As shown in Fig 3A, the reactivation of latent HIV-1 stimulated by both prostratin and CPI-203 could be effectively increased during serum starvation (by removing FBS (FBS-)) in two latently infected cells. Immunofluorescence assay identified that serum starvation caused YAP translocation from the nucleus to the cytoplasm in J-Lat A2 cells (Fig 3B). This effect might be independent of the cytotoxicity induced by serum starvation (Fig 3C). Notably, serum starvation significantly enhanced the reactivation of latent HIV-1 stimulated by a BET inhibitor CPI-203 (32.9%) in J-Lat A2 cells, whereas the effect was weak (17.1%) in J-Lat 10.6 cells (Fig 3A). The integration characteristics of the HIV-1 minigenome in J-Lat 10.6 and J-Lat A2 cells are different, and the most significant difference is in the expression of HIV-1 Tat protein (Fig 3D). Interestingly, there was a significant difference in the expression of YAP between J-Lat 10.6 and J-Lat A2 cells (Fig 3E). These results strongly indicated that there may be a possible interaction between YAP and Tat.

### YAP inhibits Tat-mediated HIV-1 transcription by binding to the Tat transactivation region

**Fig 4:**
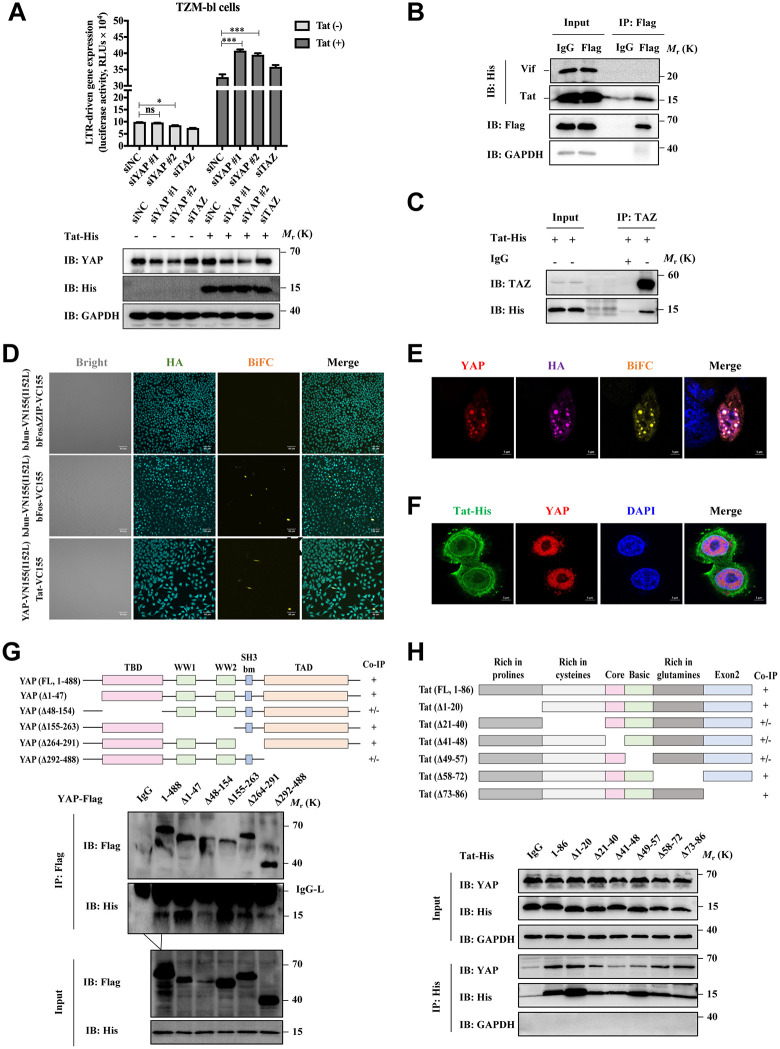
YAP binds to the Tat transactivation region. **A)** TZM-bl cells with altered YAP levels (lower) were transfected with pLVX-Tat-His or lentiviral vector, and HIV-1 LTR-driven luciferase activity was measured (upper). **B**, **C)** Co-IP analysis of the association of YAP/TAZ and Tat in HEK293T cells transfected with the indicated exogenous gene expression vectors by using a primary antibody or a relative IgG control. **D)** BiFC assay of TZM-bl cells transfected with the indicated exogenous gene expression vector with the immunostaining signals of HA (N-terminal on VC155, cyan, Alexa Fluor 405). (Scale bar, 60 μm). **E)** BiFC assay of TZM-bl cells transfected with YAP-VN155(I152L) and Tat-VC155 with the immunostaining signals of YAP (anti-YAP, red, Alexa Fluor 647) and Tat (anti-HA, purple, Alexa Fluor 555). BiFC fluorescence is shown in yellow, and nuclear staining (DAPI) is shown in blue. (Scale bar, 5 μm). **F)** Fluorescence microscopy showing the colocalization of Tat and YAP in TZM-bl cells. (Scale bar, 5 μm) **G** and **H)** Mapping of the domains that interact between YAP and Tat in HEK293T cells transfected with the indicated exogenous gene expression vector via Flag-trap **G**) or His-trap **H**) coimmunoprecipitation. Schematic diagrams of YAP or Tat truncation mutants are shown in the upper panels (“+” indicates an interaction, “−” indicates no interaction, and “+/−” indicates a weak interaction). Data were normalized to the values of the control group and are presented as the means ± SDs. Two-way ANOVA was conducted to detect significant differences (*P < 0.05, **P < 0.01, ***P < 0.001).

Here, we first detected the luciferase activity in Tat-expressing and normal TZM-bl cells treated with siYAP or siNC for 48 h by a microplate reader. The levels of Tat and YAP in those cells were confirmed by western blot assay (Fig 4A). Results showed that YAP depletion significantly promoted HIV-1 Tat-mediated LTR-driven transcription in Tat-overexpressing TZM-bl cells (Fig 4A), while no obvious effect was observed on the basal transcriptional activity of TZM-bl cells, suggesting that YAP may not directly regulate the HIV-1 LTR. To clarify this, we performed CUT&Tag-qPCR analysis and found that YAP does not directly bind to the HIV-1 LTR region (S4 Fig). Instead, co-IP assay confirmed that exogenous YAP directly bound to Tat protein in HEK293T cells, but not to HIV-1 Vif protein (Fig 4B). This interaction was further shown to be independent of TAR RNA, as it persisted in the presence of RNase A (S5A Fig). Moreover, we found that TAZ, a YAP ortholog, also binds to Tat (Fig 4C), indicating functional conservation within this regulatory axis. Together, these results position YAP as a specific transcriptional regulator of Tat rather than a direct modulator of viral promoter activity.

A BiFC assay relies on the structural complementation of two nonfluorescent fragments, VN155-I152L (N-terminal) and VC155 (C-terminal) of Venus fluorescent protein. When fused with interacting proteins, the interaction reconstitutes fluorescence by assembling those fragments. To validate the direct interaction between YAP and Tat in living cells and determine their subcellular co-localization, we generated two protein fragments by fusing VN155-I152L to the C-terminus of YAP and VC155 to the C-terminus of Tat. Here, bFos-VC155/bJun-VN155-I152L and bFosΔZIP-VC155/bJun-VN155-I152L were chosen as positive and negative controls as previously described [22]. As shown in Fig 4D, the BiFC signal of YAP-VN155-I152L/Tat-VC155 group was obviously enhanced, but weaker than that of the positive control group. Furthermore, we visualized the cellular distributions of YAP, Tat (HA-tag, N-terminal on VC155), and BiFC by an immunofluorescence staining. Both the BiFC signals and an immunofluorescence assay revealed that YAP and Tat proteins co-localized in the nucleus (Fig 4E-4F).

Considering the importance of YAP and Tat binding, we further identified the critical structural domain(s) involved in the interaction between YAP and Tat. Based on the structural analysis, we first generated five Flag-tagged deletion mutants of YAP (Fig 4G) and six His-tagged deletion mutants of Tat (Fig 4H). By a co-IP assay, YAP interacted with Tat through its TBD (amino acids 48–154) and TAD (amino acids 292–488) domains (Fig 4G). The second (amino acids 21–40), third (CD, amino acids 41–48), and fourth (BD, amino acids 49–57) domains of Tat were required for the binding with YAP (Fig 4H). Notably, the second and third domains of Tat function as trans-activation regions, which are responsible for binding transcription factors such as p65 and cyclin T1. We further tested whether YAP could disrupt the association of p65 and cyclin T1 with Tat. Results showed that the full-length YAP significantly reduced the association of p65 and cyclin T1 with Tat, while the truncated YAP mutant (∆TBD) had no similar effect (S5B, S5D Figs). These results reinforced the importance of the Tat trans-activation region and YAP TBD domain for their association.

To further verify the competitive role of YAP and cyclin T1 in Tat transactivation, we constructed a Tat mutant variant C22G (Tat^C22G^). Tat^C22G^ is a well-known mutant that is difficult to bind to cyclin T1 and therefore loses the activity of Tat transactivation. As shown in S5C Fig, the binding of Tat^C22G^ to YAP was reduced. Taken together, these findings indicate that YAP binds to the Tat transactivation region, thereby potentially suppressing the role of Tat on HIV-1 transactivation.

### YAP impaired the stability of Tat by enhancing its K33-/K48-linked polyubiquitination

**Fig 5:**
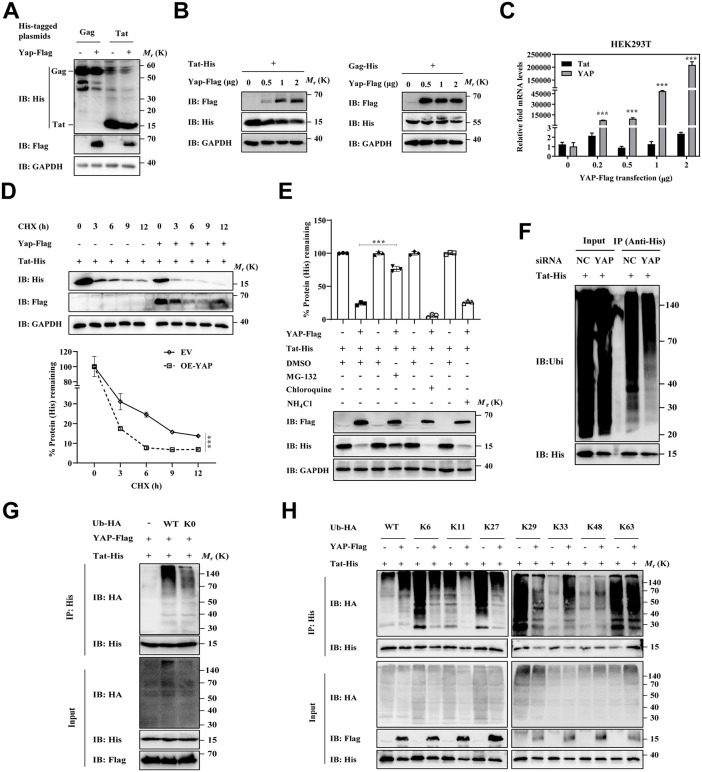
YAP impaired Tat protein stability by enhancing the K33-/K48-linked polyubiquitination of Tat. **A)** Immunoblot analysis of HEK293T cells transfected with the YAP-Flag and His-tagged Gag or Tat proteins. **B)** Immunoblot analysis of the protein expression levels of YAP in HEK293T cells transfected with increasing amounts of YAP-Flag expression plasmids together with Tat-His (left) or Gag-His (right). **C)** RT-qPCR analysis of the mRNA expression levels of YAP in HEK293T cells transfected with increasing amounts of YAP-Flag expression plasmids together with Tat-His. **D)** Immunoblot analysis of lysates of HEK293T cells co-transfected with Tat-His or YAP-Flag and treated with CHX (20 μM) at the indicated times before harvest and quantification of the band intensity of His in the upper blot normalized to the value for CHX-treated (0 h) cells. **E)** Immunoblot analysis (bottom) of lysates of HEK293T cells transfected with Tat-His together with or without YAP-Flag and treated with vehicle (DMSO), MG-132 (10 µM), chloroquine (Chlq, 100 µM), or NH_4_Cl (10 mM) for 6 h before harvest and quantification of the band intensity of His (top) in the bottom blot, normalized to the value for DMSO-treated (0 h) cells. **F)** Ubiquitination assay of exogenous Tat in HEK293T cells transfected with Tat-His and siNC or siYAP. **G)** Ubiquitination assay of exogenous Tat in HEK293T cells coexpressing Tat-His, YAP-Flag, and HA-tagged wild-type ubiquitin (Ub) or Ub-K0 mutants (all lysine residues were converted into arginine). **H)** Ubiquitination assay of exogenous Tat in HEK293T cells co-transfected with Tat-His, with or without YAP-Flag, and with HA-tagged wild-type ubiquitin (Ubi) or individual Lys^only^ ubiquitin mutants. The data were normalized to the values of the control group and are presented as the means ± SDs. To detect significant differences, two-way ANOVA was conducted in panel **C)**, and one-way ANOVA was conducted in panel **E)** (*P < 0.05, **P < 0.01, ***P < 0.001).

As shown in Fig 5A and 5B, YAP decreased the expression of Tat protein in a dose-dependent manner, but did not affect the expression of Gag protein. It is worth noting that YAP did not affect the mRNA levels of Tat (Fig 5C), indicating that YAP may promote Tat degradation at the posttranscriptional stage. Numerous studies have confirmed that YAP can affect the stability of proteins by ubiquitination [23,24]. Here, we further investigated the effect of YAP on the stability of Tat protein using a cycloheximide (CHX) chase assay. Results showed that the overexpression of YAP significantly promoted the degradation and reduced the half-life of Tat protein (Fig 5D). Furthermore, lysosome inhibitors NH_4_Cl and chloroquine were unable to inhibit the YAP-induced Tat degradation, while a proteasome inhibitor MG-132 efficiently restored Tat expression in YAP-overexpressing HEK293T cells (Fig 5E). Those results indicated that YAP impairs the stability of Tat and promotes the proteasomal degradation of Tat.

Ubiquitination is a key step in protein degradation by the ubiquitin-proteasome degradation pathway. We further evaluated the effects of YAP on the ubiquitination of the Tat protein. As shown in Fig 5F, the deletion of YAP by siRNA substantially attenuated the ubiquitination of Tat, suggesting that YAP regulates Tat ubiquitination. Different ubiquitin linkage patterns have distinct effects on cellular functions. Previous studies have confirmed that Tat tends to undergo polyubiquitination rather than multiple forms of monoubiquitination [25]. To further investigate the effect of YAP on Tat ubiquitin linkage patterns, a Ub-K0 plasmid (a multipoint mutant in which all seven lysine residues were replaced by arginine to prevent the formation of ubiquitin chains) and Ub-Kx plasmids (a multipoint mutant in which six lysine residues were replaced by arginine) were constructed. Compared with the wild-type (WT) ubiquitin vector group, the YAP-induced Tat ubiquitination in the ubiquitin mutant (Ub-K0) group was obviously reduced, indicating that YAP induced Tat polyubiquitination (**Fig 5G**). By an immunoblotting assay, YAP specifically promoted K33- and K48-linked Tat polyubiquitination, especially on K33-linked polyubiquitination (**Fig 5H**). It has been proven that K48-linked Tat polyubiquitination requires the initiation of K33 modification, which may serve as an acceptor site for chain formation [25]. Notably, YAP inversely reduced the K6-, K11-, K27-, and K29-linked Tat polyubiquitination. However, YAP increased the total polyubiquitination level of Tat, indicating that K33- and K48-linked Tat polyubiquitination may play a dominant role in YAP-induced Tat ubiquitination. Due to the dependence of Tat ubiquitination on lysine residues, we further investigated which lysine residues are necessary for Tat ubiquitination. Results revealed that none of these Tat lysine-to-arginine point mutants inhibited Tat ubiquitination **(****S6 Fig**). Collectively, YAP promotes K33/K48-linked polyubiquitination at non-single acceptor lysine sites, thereby controlling Tat degradation.

### Conserved lysine residues 28, 29, and 41 of Tat may function as ubiquitin acceptor sites for proteasomal degradation

**Fig 6:**
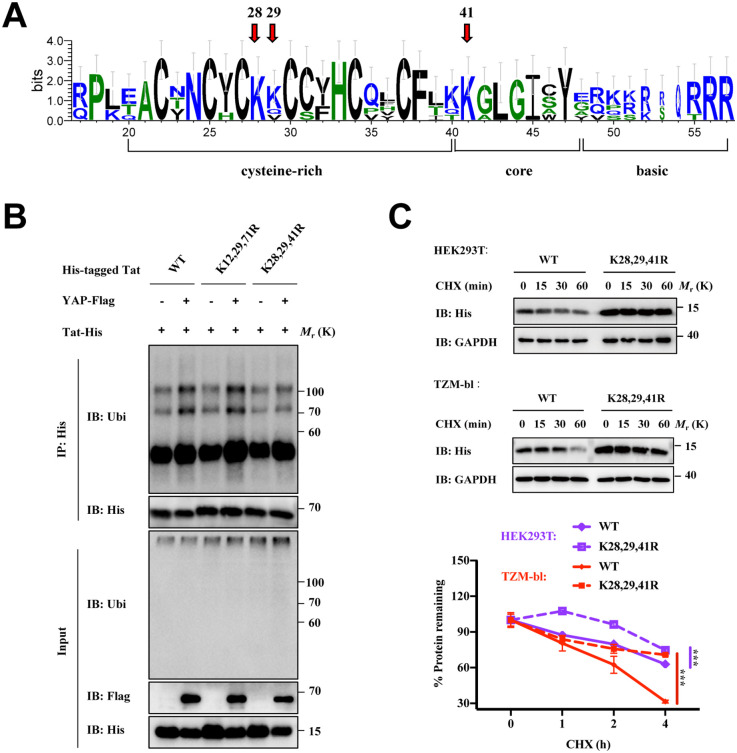
Conserved lysine residues 28, 29, and 41 of the Tat protein might function as ubiquitin acceptor sites for proteasomal degradation. **A)** Sequence logos showing conserved regions of HIV and SIV Tat were computed from the sequence alignments, which were extracted from the UniProtKB/Swiss-Prot database (HIV-1: P04608.1, HIV-2: P18044.1, SIVcpz: I2BF62, SIVagm: Q88106, SIVsmm: Q88106, SIVagm: A0A2P1DQ03). The figure was generated via WebLogo 3. **B)** Ubiquitination assay of His-tagged wild-type Tat and Lys-to-Arg mutants (K12,29,71R and K28,29,41R) in HEK293T cells co-transfected with YAP-Flag or the vector. **C)** CHX assay of His-tagged wild-type Tat and Lys-to-Arg mutant (K28,29,41R) in HEK293T cells (upper) and TZM-bl cells (middle). The band intensity of His in these blots was normalized to the value for CHX-treated (0 h) cells and is shown (bottom). Data were normalized to the values of the control group and are presented as the means ± SDs. Two-way ANOVA was conducted to detect significant differences (***P < 0.001).

Although Tat can tolerate up to 40% of sequence variations without losing its bioactivity, there are several conserved sequences in the critical functional regions of Tat in both HIV and simian immunodeficiency virus (SIV) [26]. Based on sequence alignments, the cysteine-rich fragments, core domains, and basic domains of HIV/SIV Tat are relatively conserved regions (Fig 6A). Interestingly, these regions are also necessary for the binding of HIV-1 Tat and YAP. Previous studies have reported that the transcriptional activity of three lysine Tat variants containing lysine residues 28 and 41 is almost identical to wild-type Tat, while mutations at K12, K29, and K71 reduced Tat ubiquitination [25]. Here, we constructed Tat^K28,29,41R^ and Tat^K12,29,71R^ mutants. As shown in Fig 6B, only Tat^K28,29,41R^ mutant partially inhibited YAP-mediated ubiquitination of Tat. Compared with that of wild-type Tat, the stability of the Tat^K28,29,41R^ mutant was obviously enhanced in both HEK293T and TZM-bl cells by a CHX chase assay (Figs 6C and S7A). Research has shown that the K41 mutation of Tat could disrupt the stability of Tat and reduce Tat transcriptional activity. Interestingly, Tat^K28,29,41R^ mutant unexpectedly restored some of the transcriptional activity lost by Tat^K41R^ mutant, which may be attributed to the restoration of Tat protein levels (S7B Fig). Furthermore, only Tat^K28,29,41R^ mutant losed its promoting activity on viral infection (S7C Fig). Taken together, the conserved lysine residues K28, K29, and K41 in Tat may function as ubiquitin acceptor sites for the ubiquitination and proteasomal degradation of Tat.

### UHRF1, as an E3 ubiquitin ligase, triggers YAP-mediated Tat ubiquitination

**Fig 7:**
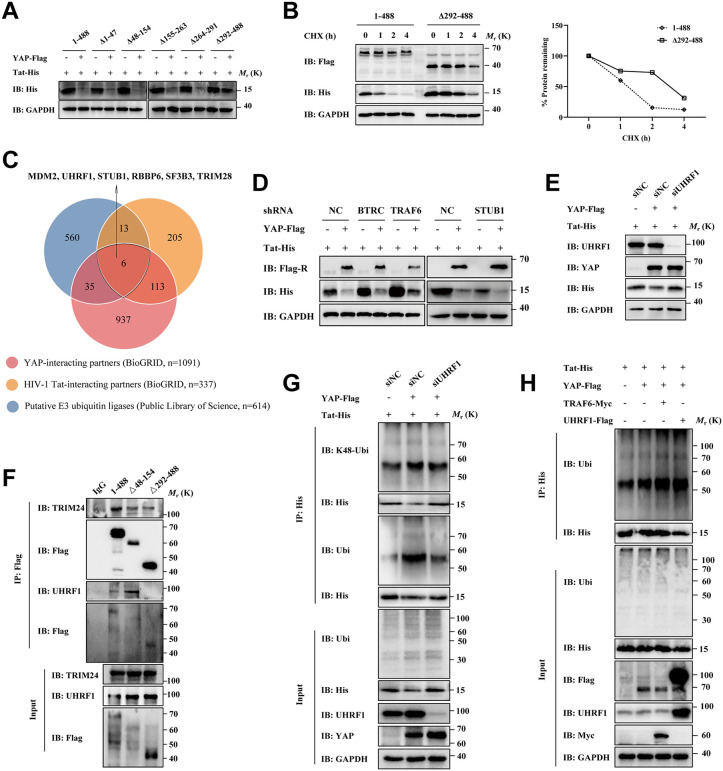
UHRF1 triggers YAP-mediated Tat ubiquitination as an E3 ubiquitin ligase. **A)** Immunoblot analysis of lysates of HEK293T cells co-transfected with Flag-tagged full-length YAP or truncated mutants or vector and Tat-His for 48 h before harvest. **B)** CHX assay of His-tagged Tat in HEK293T cells transfected with plasmids encoding Flag-tagged full-length YAP or the truncated mutants YAP^Δ292-488^ and Tat-His, and quantification of the band intensity of His in the right blot normalized to the value for CHX-treated (0 h) cells. **C)** Venn diagram showing the overlap of the proteins among datasets obtained from BioGRID and the Public Library of Science. **D)** Immunoblot analysis of lysates of lentivector-transduced HEK293T cells (shNC, shBTRC, shTRAF6, and shSTUB1) co-transfected with Tat-His and YAP-Flag or vector for 36 h before harvest. **E)** Immunoblot analysis of lysates of HEK293T cells co-transfected with Tat-His, YAP-Flag, or vector, and siNC or siUHRF1. **F)** Flag-trap coimmunoprecipitation assay of the association of Flag-tagged YAP with UHRF1 and TRIM24 in HEK293T cells transfected with Flag-tagged full-length YAP or the indicated truncated mutants for 48 **h. G)** Ubiquitination assay of His-tagged Tat in small interfering RNA-transfected HEK293T cells (siNC and siUHRF1) co-transfected with Tat-His and YAP-Flag or vector for 48 **h. H)** Ubiquitination assay of His-tagged Tat in HEK293T cells co-transfected with the indicated plasmids encoding Tat-His, YAP-Flag, TRAF6-Myc, and UHRF1-Flag for 48 **h.** Data are normalized to the values of the control group and are presented as the means ± SDs.

Due to the lack of E3 ubiquitin ligase activity in YAP, YAP may induce Tat ubiquitination by recruiting YAP-associated enzymes. Here, we first evaluated the effects of five truncated forms of YAP on Tat degradation by immunoblotting and a CHX chase assay. As shown in Fig 7A and 7B, the deletion mutation of YAP TAD domain (YAP Δ292–488) effectively weakened YAP-mediated Tat degradation, indicating that the YAP TAD domain might be the binding domain of the E3 ubiquitin ligase. Subsequently, we screened the specific E3 ligases that may interact with both YAP and Tat using the BioGRID database and a putative E3 ubiquitin ligase library [27]. The Venn diagram displayed six overlapping proteins in the dataset, including TRIM28, SF3B3, RBBP6, STUB1, UHRF1, and MDM2 (Fig 7C). The latest literature confirmed that a novel E3 ubiquitin ligase, TRAF6, also interacts with both YAP and Tat, although it has not yet been included in the database (BioGRID Version 4.4.233) [28, 29]. To identify the E3 ligase that specifically binds to the YAP TAD domain, HEK293T cells transfected with Flag-YAP (FL, ∆TBD, or ∆TAD) were used for immunoprecipitation-mass spectrometry (IP-MS) analysis. Four E3 ubiquitin ligases (KCTD20, KLHL8, ZFPL1, and MID2) failed to bind to the YAP ∆TAD mutant revealed by IP-MS assay (S8A Fig), but none of them affected the transcriptional activity or stability of the Tat protein (S8B, S8C Figs). The data from IP-MS analysis revealed that YAP could bind to TRIM28, SF3B3, and RBBP6, but these ligases were ruled out because YAP truncation did not affect their recruitment. MDM2 was also excluded because it did not influence Tat degradation [30]. Therefore, we identified the key E3 ligases among STUB1, UHRF1, and TRAF6 by a rescue assay. Our results confirmed that the depletion of STUB1 or TRAF6 was not able to rescue the expression of Tat protein in the presence of YAP (Fig 7D), while the depletion of UHRF1 could effectively rescue that of Tat (Fig 7E). Using a co-immunoprecipitation (co-IP) assay, we showed that UHRF1 specifically interacts with the TAD domain of YAP (Fig 7F). This interaction is mediated by the RING domain of UHRF1 (S9 Fig). Our previous results showed that UHRF1 functions as an E3 ubiquitin ligase and mediates K48-linked ubiquitination and proteasomal degradation of Tat through its RING domain [31]. Here, we investigated whether UHRF1 impacts YAP-mediated Tat ubiquitination. Our findings verified that Tat ubiquitination in the presence of YAP could be clearly decreased by UHRF1 reduction (Fig 7G). Consistently, UHRF1 overexpression significantly increased YAP-induced Tat ubiquitination (Fig 7H). Collectively, these results suggest that YAP-induced Tat degradation may be dependent on the recruitment of the E3 ligase UHRF1. Future studies aimed at elucidating the precise spatiotemporal dynamics of these complex assemblies will be valuable.

## Discussion

The Hippo pathway plays a role in innate immune responses, including antibacterial and antiviral immunity, as well as macrophage polarization. As an effector of the Hippo pathway, YAP is closely related to the pathogenesis of viruses [10–18], but its role in HIV-1 remains unclear. An analysis of an mRNA microarray dataset (GSE140713) revealed that YAP gene expression was significantly downregulated in the AIDS patient group. We thus detected the effect of YAP on HIV-1 infection and HIV-1 latency. Our results reveal that YAP deficiency enhances HIV-1 infection and reactivation, whereas YAP overexpression decreases that. Both YAP and its ortholog TAZ negatively regulated HIV-1 replication. As we all know, serum starvation triggers YAP signaling and leads to YAP protein levels down. Our results confirmed that serum starvation led to the translocation of YAP from the nucleus to the cytoplasm, a signal for YAP degradation, which enhanced the reactivation of latent HIV-1 by LRAs. Taken together, these findings indicate that YAP may play a negative regulatory role in HIV-1 transcription and infection.

Interestingly, serum starvation strongly increased the ability of LRAs to reactivate latent HIV-1 in J-Lat A2 cells but not in J-Lat 10.6 cells. J-Lat A2 cells harboring the HIV-1 minigenome “LTR-Tat-IRES-GFP” presented high levels of Tat expression, along with a marked increase in YAP mRNA. Given that YAP functions as a transcriptional coactivator by interacting with various transcription factors, such as TEAD1/2/3/4, RUNX1/2, p65/p73, and ErbB4 of host infector [32,33], and TAX of HTLV-1 [17]. We hypothesized that, similar to its interaction with HTLV-1 TAX, YAP may connect to the HIV-1 transactivator Tat. Numerous studies have shown that Tat controls the latency and transcription of HIV-1 by recruiting p-TEFb and promoting transcription [34–36]. Here, we demonstrated that YAP deficiency potentiates Tat-mediated HIV-1 LTR transcription, potentially through its dissociation from Tat. Notably, we found that YAP binds to Tat through multiple domains, including the TBD and TAD domains. Confirming this interaction at the endogenous level in primary latency models remains an important future direction. Interestingly, the enhancement of LRA-induced reactivation by YAP knockdown was comparable in both J-Lat A2 and J-Lat 10.6 cells. Previous reports have indicated that activation of the NF-κB pathway can trigger the nuclear export of YAP, thereby facilitating the nuclear import of p65 [37]. The nuclear export of YAP induced by serum starvation could therefore favor Tat-p65-driven HIV-1 LTR transcription, which may explain reactivation in ACH2 cells despite their defective TAR domain. This points to the therapeutic potential of targeting YAP’s nuclear localization signals.

Tat is a small and highly flexible protein that is typically composed of 86–101 amino acids, depending on the viral subtype. Owing to its rapid degradation, Tat has a relatively short half-life, which is partly regulated by its posttranslational modifications, such as ubiquitination [38,39]. YAP has been shown to influence the stability of certain proteins, such as NLRP3 and TRAF6, through ubiquitination [28,40]. Interestingly, we found that YAP specifically promoted Tat degradation by inducing K33- and K48-linked ubiquitination of Tat. In general, all polyubiquitin linkages in addition to Lysine 63, generally result in proteasomal degradation [41]. Tat can be modified with various polyubiquitin chains, and its ubiquitination is lysine-dependent. Notably, studies have shown that ubiquitin variants with single lysine K27, K29, and K33 residues can restore full or partial Tat polyubiquitination, whereas it seems that only ubiquitin variants with a single lysine point mutation, K33R, can diminish Tat polyubiquitination [25]. Collectively, these findings suggest that the ubiquitin Lys 33 residue plays a key role in the ubiquitination-proteasome degradation of Tat.

The flexibility of the ubiquitin attachment sites on Tat is an intriguing theme. Previous studies and our study have shown that Tat undergoes ubiquitination at multiple redundant lysine residues [25]. Only lysines K12, K29, and K71 were identified as ubiquitinated sites of Tat by an MS assay. The nonproteolytic role of polyubiquitination at lysine 71 has been fully demonstrated, but there is limited research on other lysine residues [42]. We found that the ubiquitin variants K12/29/71R were unable to diminish the induction of Tat polyubiquitination by YAP, but the variant mutant at highly conserved lysines K28, K29, and K41 (K28/29/41R) partially inhibited YAP-mediated Tat polyubiquitination. Consistent with previous studies, the ubiquitination levels of three lysine Tat variants (K28, K29, and K41) were close to those of wild-type Tat [25]. These results indicated that lysines K28, K29, and K41 may serve as acceptor sites mediating the ubiquitination and proteasomal degradation of Tat. As expected, we found that the triple mutant of Tat (K28/29/41R) was less susceptible to degradation than the point mutant Tat (K41R) or even wild-type Tat. Mutation from lysine 41 to arginine or alanine (K41R/A) disrupts the structure of Tat, leading to a decrease in overall protein accumulation [38,43]. However, although the K41R mutation and complete lysine deletion (∆K) mutants significantly inhibited Tat activity, the triple mutant K28/29/41R surprisingly restored some of the activity lost in the K41R mutant, possibly due to the recovery of Tat protein levels. Despite the presence of multiple redundant acceptor lysines, these results further confirm the critical roles of the conserved lysine residues K28, K29, and K41 in Tat ubiquitination and proteasomal degradation. Also, the role of these conserved lysine residues of HIV-2/SIV Tat for their ubiquitination and proteasomal degradation is worthy of further determination.

Owing to the lack of E3 ubiquitin ligase activity in YAP, YAP may induce Tat ubiquitination by recruiting YAP-associated enzymes. Previous studies have shown that the TBD domain of YAP interacts with transcription factors such as TEAD and AXIN, whereas the TAD domain binds to certain E3 ubiquitin ligases, such as TRAF6 and BTRC [32]. Here, we found that YAP regulates Tat ubiquitination and degradation through its TAD domain. To identify the specific E3 ligase involved, we performed an IP-MS screen. The results revealed four E3 ubiquitin ligases that may specifically bind to the YAP TAD domain, but none of them affected Tat transcriptional activity or stability. This screening prompted us to prioritize UHRF1, a YAP-associated E3 ligase identified from the BioGRID database, for subsequent analysis. We determined that the TAD domain recruits the E3 ubiquitin ligase UHRF1 to mediate Tat degradation. UHRF1 was identified in previous work as a repressor of HIV-1 latency because of its role in HIV-1 epigenetic silencing or Tat ubiquitination and degradation [31,44,45]. It is noteworthy that UHRF1 could stabilize the YAP protein [46]. The concrete control network of the cooperation of UHRF1 and YAP on Tat ubiquitination and degradation is worthy of further determination. Due to the focused scope of our study, we did not systematically examine other known regulators of Tat stability and activity, such as PJA2 or the CUL4B-DDB1-DCAF1 complex [47–49]. The potential cross-talk or complementary functions between these pathways and the YAP-UHRF1 axis represent an important area for future investigation. In a word, the mediating role of UHRF1 in YAP-induced Tat degradation determined in this study further highlights the role of UHRF1 in HIV-1 cure strategies.

**Fig 8:**
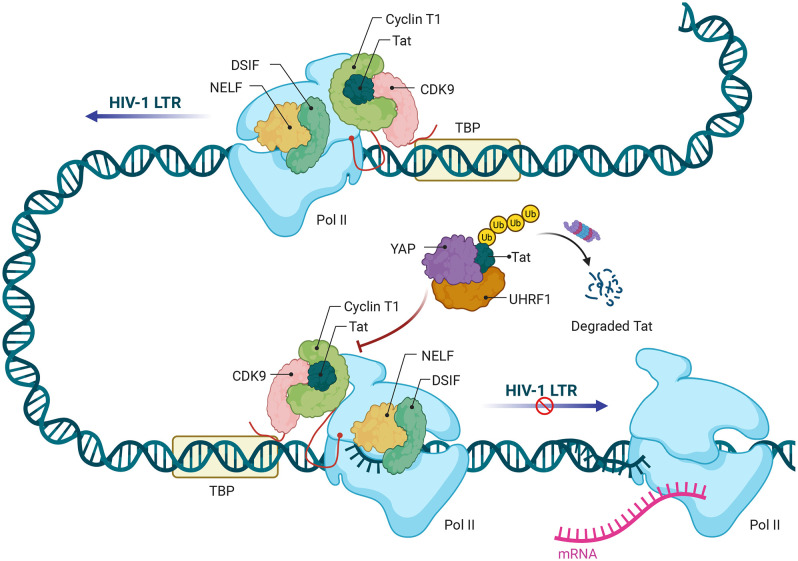
Schematic illustration of how YAP suppresses HIV-1 transcription. HIV-1 Tat recruits host cyclin T1 and CDK9 (positive transcription elongation complex b, p-TEFb) to activate RNA polymerase II (Pol II) for transcriptional elongation in the HIV-1 LTR. YAP recruits the E3 ligase UHRF1 to bind with Tat, followed by the ubiquitination of Tat at the conserved lysines K28, K29, and K41. Tat thus undergoes K33- and K48-linked polyubiquitination and proteasomal degradation, thereby inactivating RNA polymerase II (Pol II) to pause HIV-1 transcription. Created in BioRender. zhou, **c.** (2026) https://BioRender.com/0p5u8o6.

In summary, our work defines a previously unrecognized pathway whereby YAP recruits the E3 ligase UHRF1 to target HIV-1 Tat for ubiquitination and degradation, ultimately establishing YAP as a host factor that suppresses viral transcription and promotes latency (**Fig 8**). We demonstrated that the conserved lysines K28, K29, and K41 of Tat are critical for its ubiquitination and proteasome-mediated degradation. The pharmacological inhibition of YAP (e.g., using the YAP-TEAD inhibitor Verteporfin) presents a promising avenue for synergizing with existing LRAs to reactivate latent proviruses, warranting further exploration. It is noteworthy that while YAP is a known regulator of cellular Pol II pause-release, its dominant role in HIV-1 suppression appears to be the targeted elimination of Tat, as our data indicate no direct YAP binding to the viral promoter. This Tat-centric mechanism may be further reinforced by YAP’s broader transcriptional network, such as its interplay with the host restriction factor BRD4, highlighting a multi-layered control of viral latency.

## Materials and methods

### Ethics statement

The study was reviewed and approved by the Ethics Committees of Nanfang Hospital (reference no. NFEC-2021–448). All donors provided written informed consent.

### Cells

Jurkat, J-Lat 10.6, J-Lat A2, U1, ACH2, U87-CD4-CXCR4, MT-2, and TZM-bl cells were obtained from the National Institutes of Health AIDS Research and Reference Reagent Program (NIH AIDS Reagent Program). HEK293T cells were purchased from the American Type Culture Collection (ATCC). The cells were cultured in RPMI 1640 medium (Gibco) or Dulbecco’s modified Eagle’s medium (DMEM, Gibco) supplemented with 10% fetal bovine serum (FBS) and 1% penicillin/streptomycin (Invitrogen) in a 37°C incubator containing 5% CO_2_.

Peripheral blood mononuclear cells (PBMCs) were isolated from the blood of healthy donors or HIV-1-infected patients at Nanfang Hospital as previously described [50]. In general, the isolated cells were treated with IL-2 (50 IU/mL) and PHA (5 μg/mL) before HIV-1 infection. Primary CD4^+^ T cells were isolated from PBMCs via an EasySep Human CD4-Positive Selection Kit II (STEMCELL Technologies). The characteristics of the HIV-1-infected individuals receiving cART are listed in Table 1.

**Table 1:** Characteristics of cART-treated HIV-1-infected individuals in this study.

Number	Sex	Age (yrs)	CD4 counts (cells/mm³)	Time under ART (yrs)	Viral load (HIV RNA copies/ml plasma)
A00384	M	27	1070	5.5	<100
A00573	M	56	678	5.1	<100
A01159	M	44	543	3.8	<100

### Plasmids

pLV3-YAP-mCherry, HA-Ubiquitin (K6, K11, K27, K29, K33), pLV3-STUB1-shRNA1-CopGFP, and the expression vectors Tet-pLKO-puro, pLKO.1-mCherry-puro, pLV3-CMV-mCherry-puro, and pBiFC-VN155 (I152L) were obtained from MiaoLingBio (China). pBiFC-bJunVN155 (I152L), pBiFC-bFos∆ZipVC155, and pBiFC-bFosVC155 were purchased from GLS Biology (China). The pBiFC-VC155 vector was kindly provided by Prof. Xingang Yao of Southern Medical University (SMU), China. The expression vectors for YAP (pcDNA3.1 and pLVX vectors) were kindly provided by Prof. Le Yu of SMU. The HA-Ubiquitin (WT, K48, K63), HIV-1 Tat, Vif, Gag, and UHRF1 plasmids were prepared as previously described [31]. All mutations of Tat (∆1–20, ∆21–40, ∆41–48, ∆49–57, ∆58–72, ∆73–86, K12R, K19R, C22G, K28R, K29R, K41R, K50R, K51R, K71R) were inserted into pLVX vectors with His tags. The YAP deletion mutants (∆1–47, ∆48–154, ∆155–263, ∆264–291, and ∆292–488) were subsequently subcloned and inserted into a Flag-tagged pcDNA3.1 vector. All sequences are listed in **Table 2**.

**Table 2:** Construct primers.

Primer name	Template	Primer Sequence (5’-3’)
pcDNA-YAP-FL Fwd	pcDNA-YAP-Flag	TAGTCCAGTGTGGTGGAATTCATGGATTATAAAGATGATGATGATAAAGATC
pcDNA-YAP-FL Rev	pcDNA-YAP-Flag	GGTTTAAACGGGCCCTCTAGACTATAACCATGTAAGAAAGCTTTCTTTATC
pcDNA-YAP-Δ1–47 Fwd	pcDNA-YAP-Flag	CCGGAATTCATGGATTATAAAGATGATGATGATAAACCCGCCGGGCATCAGATC
pcDNA-YAP-Δ48–154 1Rev	pcDNA-YAP-Flag	TGTCGAAGATGCTGAGCTGTGGGTGCCTGCGGCGCCGC
pcDNA-YAP-Δ48–154 2Fwd	pcDNA-YAP-Flag	ACAGCTCAGCATCTTCGACAGTC
pcDNA-YAP-Δ155–263 1Rev	pcDNA-YAP-Flag	TGGTTCATGGCAAAACGAGGGGGTGTAGCTGCTGGGCC
pcDNA-YAP-Δ155–263 2Fwd	pcDNA-YAP-Flag	CCTCGTTTTGCCATGAACCA
pcDNA-YAP-Δ264–291 1Rev	pcDNA-YAP-Flag	CCATGACGCCGTCAAGCCTTGGGTCTAGCCA
pcDNA-YAP-Δ264–291 2Fwd	pcDNA-YAP-Flag	AAGGCTTGACGGCGTCATGGGTGGCAGC
pcDNA-YAP-Δ292–488 Rev	pcDNA-YAP-Flag	GGTTTAAACGGGCCCTCTAGACTATCCCTGTGGGCTCTGGG
pLVX-Tat-FL Fwd	pLVX-Tat-His	GGATCTATTTCCGGTGAATTCATGGAGCCAGTAGATCCTAGACTAGAG
pLVX-Tat-FL Rev	pLVX-Tat-His	CAGGGCCAGGTTTCCGGGCCCTTAGTGGTGATGGTGATGATGTTCC
pLVX-Tat-Δ1–20 Fwd	pLVX-Tat-His	GGATCTATTTCCGGTGAATTCATGTGTACCAATTGCTATTGTAAAAAGTGTT
pLVX-Tat-Δ21–40 1Rev	pLVX-Tat-His	ACAAGCAGTTTTAGGCTGACTTCCTG
pLVX-Tat-Δ21–40 2Fwd	pLVX-Tat-His	GTCAGCCTAAAACTGCTTGTTTCATAACAAAAGCCTTAGGC
pLVX-Tat-Δ41–48 1Rev	pLVX-Tat-His	CGCTTCTTCCTAACTTGGCAATGAAAGCAACACT
pLVX-Tat-Δ41–48 2Fwd	pLVX-Tat-His	TGCCAAGTTAGGAAGAAGCGGAGACAGCG
pLVX-Tat-Δ49–57 1Rev	pLVX-Tat-His	TTCTGATGAGCGCCATAGGAGATGCCTAAGGC
pLVX-Tat-Δ49–57 2Fwd	pLVX-Tat-His	TCCTATGGCGCTCATCAGAACAGTCAGACTCATCA
pLVX-Tat-Δ58–72 1Rev	pLVX-Tat-His	TTGGGAGGTGGGTCTTCGTCGCTGTCTCCGC
pLVX-Tat-Δ58–72 2Fwd	pLVX-Tat-His	GACGAAGACCCACCTCCCAACCCCGA
pLVX-Tat-Δ73–86 Fwd	pLVX-Tat-His	CCGGAATTCATGATGGAGCCAGTAGATCCTA
pLVX-Tat-Δ73–86 Rev	pLVX-Tat-His	CGCGGGCCCTTAGTGGTGATGGTGATGATGTTGCTTTGATAGAGAAGC
pBiFC-YAP-VN155 Fwd	pcDNA-YAP-Flag	CCGGAATTCTAGATCCCGGGCAGCAGCCG
pBiFC-YAP-VN155 Rev	pcDNA-YAP-Flag	GGGGTACCTAACCATGTAAGAAAGCTTTC
pBiFC-Tat-VC155 Fwd	pLVX-Tat-His	CCGGAATTCCGGAGCCAGTAGATCCTAGA
pBiFC-Tat-VC155 Rev	pLVX-Tat-His	GGGGTACCTTCCTTCGGGCCTGTCGGGTC
pRK5-Ubiquitin-K0 Fwd	pRK5-Ubiquitin-K6	ATCTTCGTGAgGACCCTGACTGGTAGGACCATCA
pRK5-Ubiquitin-K0 Rev	pRK5-Ubiquitin-K6	AGGGTCcTCACGAAGATCTGCATGGTCGACCC
pLVX-Tat-K12R Fwd	pLVX-Tat-His	AgGCATCCAGGAAGTCAGCCTAAAACTGCTTG
pLVX-Tat-K12R Rev	pLVX-Tat-His	TGACTTCCTGGATGCcTCCAGGGCTCTAGTCTAGGATCTACT
pLVX-Tat-K28R Fwd	pLVX-Tat-His	GCTATTGTAgAAAGTGTTGCTTTCATTGCCAA
pLVX-Tat-K28R Rev	pLVX-Tat-His	ACACTTTcTACAATAGCAATTGGTACAAGCAGTT
pLVX-Tat-K41R Fwd	pLVX-Tat-His	TAACAAgAGCCTTAGGCATCTCCTATGGCAGG
pLVX-Tat-K41R Rev	pLVX-Tat-His	GCCTAAGGCTcTTGTTATGAAACAAACTTGGCAATG
pLVX-Tat-K50R Fwd	pLVX-Tat-His	TCCTATGGCAGGAgGAAGCGGAGACAGCGACGA
pLVX-Tat-K50R Rev	pLVX-Tat-His	TTCcTCCTGCCATAGGAGATGCCTAAGGCTTT
pLVX-Tat-K51R Fwd	pLVX-Tat-His	TCCTATGGCAGGAAGAgGCGGAGACAGCGACGAAGA
pLVX-Tat-K51R Rev	pLVX-Tat-His	cTCTTCCTGCCATAGGAGATGCCTAAGGCTTT

The shRNAs targeting YAP, BTRC, and TRAF6 were subsequently cloned and inserted into the pLKO.1-mCherry-puro vector and/or the Tet-pLKO-puro vector. The sequences are listed in Table 3. HIV-1 Tat-K85R/ -K12,29,71R/ -K28,29,41R were synthesized and inserted into pLVX vectors with a C-terminal 6 × His tag.

**Table 3:** Oligonucleotide sequences of shRNAs or siRNAs.

shRNAs or siRNAs	shRNA-ID	Sequence (5’-3’)
YAP #1	TRCN0000300282	CCCAGTTAAATGTTCACCAAT
YAP #2	TRCN0000107266	GCCACCAAGCTAGATAAAGAA
TRAF6	TRCN0000007348	GCCACGGGAAATATGTAATAT
BTRC	TRCN0000006543	GCGTTGTATTCGATTTGATAA
siYAP #1	Not applicable	GACAUCUUCUGGUCAGAGA
siYAP #2	Not applicable	CUGCCACCAAGCUAGAUAA
siTAZ	Not applicable	AGGUACUUCCUCAAUCACA
siUHRF1	Not applicable	GCCAUACCCUCUUCGACUA
siNC	Not applicable	UUCUUCGAACGUGUCACGU

### Reagents

CPI-203 and doxycycline were purchased from MedChemExpress. MG-132, dithiothreitol (DTT), cycloheximide (CHX), and phytohemagglutinin (PHA) were purchased from Selleck Chemicals. RNase A was purchased from New England Biolabs, and Prostratin was obtained from Sigma. TNF-α and IL-2 were purchased from Sino Biological, Inc. SAHA was obtained from Topscience. PEI MAX (for DNA transfection) was purchased from Polysciences. CALNP RNAi (for siRNA transfection) was obtained from D-Nano Therapeutics.

Rabbit anti-HA/ -YAP/ -TAZ/ -GAPDH/ -ubiquitin and normal IgG antibodies were purchased from Cell Signaling Technology. Mouse anti-His tag/-normal IgG and rabbit anti-Flag antibodies were obtained from Proteintech. Mouse anti-UHRF1 antibodies were purchased from Santa Cruz. Rabbit anti-K48-linked specific ubiquitin and mouse anti-Myc tag antibodies were obtained from Abmart. A mouse anti-Flag-M2 tag was obtained from Sigma-Aldrich. An antibody specific to p24 (183-H12-5C, mouse) was obtained from the NIH AIDS Reagent Program.

### Lentivirus production and infection

The lentiviral vectors pLKO- and pLV3 expressing target genes or shRNA were co-transduced with psPAX2 and pMD2.G into HEK293T cells to produce lentiviral particles via PolyJet (SignaGen) according to the manufacturer’s instructions. After 48 h of transfection, the viral particles were harvested and filtered through a 0.45 μM filter unit.

To obtain stably expressed cell lines, the cells were spinoculated with lentivirus at 1,000 × g for 90 min and cultured at 37°C for 10 h. After the supernatants were removed, the cells were cultured for 24 h, followed by puromycin selection. Three days postselection, the supernatants of the infected cells were replaced with fresh RPMI-1640 medium, and the infected cells were cultured for another 2–7 days.

### HIV-1-infected individual samples

CD4^+^ T cells isolated from three HIV-1-infected patients were spinoculated with YAP-targeting shRNA in the presence of polybrene at 1,000 × g for 90 min. After an additional 10 h of incubation, the supernatants were replaced with fresh medium, and the cells were further incubated with IL-2 (50 IU/mL) at 37°C for 24 h. After selection with puromycin for 3 days, the knockdown efficiency of the cells was confirmed by immunofluorescence (mCherry) and RT-qPCR. Then, the cells were stimulated with TNF-α (10 ng/mL) for another 72 h. Total mRNA was extracted and detected via real-time quantitative PCR (RT-qPCR).

### RT-qPCR

The RT-qPCR assay was performed as described previously [51]. Briefly, total cellular RNA was extracted via a Super-Fast Pure Cell RNA Isolation Kit (Vazyme), and cDNA was synthesized via HiScript IV RT Super Mix (Vazyme). RT‒qPCR was performed via the use of ChamQ Universal SYBR qPCR Master Mix (Vazyme) on a LightCycler 480 system (Roche). The sequences of the primers are shown in Table 4. The amplifications were performed following the manufacturer’s instructions. The expression level was normalized to that of GAPDH. The levels of gene expression were analyzed via the delta‒delta CT (2^−ΔΔCT^) method.

**Table 4:** RT-qPCR Primers.

Primer name		Primer Sequence (5’-3’)
CTGF	forward	CCAATGACAACGCCTCCTG
	reverse	TGGTGCAGCCAGAAAGCTC
YAP	forward	GATCCCTGATGATGTACCACTGCC
	reverse	GCCATGTTGTTGTCTGATCGTTGTG
GAPDH	forward	CTCCTGCACCACCAACTGCT
	reverse	GGGCCATCCACAGTCTTCTG
Pro	forward	TGGGAGCTCTCTGGCTAACT
	reverse	TGCTAGAGATTTTCCACACTGA
Int	forward	GTAATACCCATGTTTTCAGCATTATC
	reverse	TCTGGCCTGGTGCAATAGG
Dis	forward	GAGAACTCAAGATTTCTGGGAAG
	reverse	AAAATATGCATCGCCCACAT
Initiation	forward	GTTAGACCAGATCTGAGCCT
	reverse	GTGGGTTCCCTAGTTAGCCA

### Cleavage Under Targets & Tagmentation (CUT & Tag) followed by qPCR

The CUT&Tag assay was conducted following the manufacturer’s guidelines provided in the Hyperactive Universal CUT&Tag Assay Kit for Illumina (Vazyme). HEK293T cells were first transfected with pGL3–5’LTR-luc along with either an empty vector or a YAP-Flag construct and incubated for 48 h. Subsequently, cells were harvested and quantified, with 1 × 10⁶ cells used per CUT&Tag reaction. Genomic cleavage was carried out using pA-Tn5 transposase. For quantitative assessment, CUT&Tag products were processed according to the CUT&Tag Stop Buffer protocol for qPCR (Vazyme). Quantitative PCR was then performed using ChamQ Universal SYBR qPCR Master Mix (Vazyme) on a LightCycler 480 instrument (Roche). Primer sequences are provided in S1 Table. All amplification steps adhered to the kit instructions. DNA spike-in was used for normalization, and relative quantification of gene expression was calculated using the 2^−ΔΔCT^ method.

### An HIV-1 infection assay

PBMCs, Jurkat, and TZM-bl cells were infected with replication-competent HIV-1_IIIB_ strains for the indicated durations. After 12 h of preinfection, the culture medium was replaced with fresh medium, and the cells were further cultured for the indicated periods. Then, the cells or the supernatants were lysed with lysis buffer or 5% Triton X-100 at 4°C. The luciferase activity or p24 antigen activity of the lysates was measured using a luciferase kit (Promega) or an ELISA, as described previously [52].

### Immunoblotting and coimmunoprecipitation (co-IP)

Cells transfected with YAP siRNA or off-target controls were seeded in a 6-well plate and incubated for 48 h. After 2 washes, the cells were lysed with RIPA buffer supplemented with 1 mM phenylmethanesulfonyl fluoride (Beyotime) and protease inhibitor cocktail (Beyotime) for 30 min on ice. The cell lysates were separated via sodium dodecyl sulfate‒polyacrylamide gel electrophoresis (SDS-PAGE) and then transferred to polyvinylidene fluoride membranes (Millipore). Specific primary antibodies were used, followed using an IgG-peroxidase-conjugated secondary antibody. Then, the blots were observed using chemiluminescent detection reagents (Millipore). Grayscale values were measured via ImageJ software.

For coimmunoprecipitation, cells in 10-cm culture plates were lysed in 600 μL of IP buffer (Beyotime) supplemented with 1 mM phenylmethylsulfonyl fluoride and protease inhibitor cocktail. The cell lysates were subsequently centrifuged at 12,000 × g for 15 min, after which the clarified supernatants were incubated with primary antibodies conjugated to BeyoMag Protein A + G Magnetic Beads (Beyotime) at room temperature. After 2 h of incubation, the beads were washed three times, and the pellet was resuspended in 40 μL of 2 × SDS-PAGE sample buffer and then analyzed by immunoblotting. A total of 10% of the lysates was used as the input.

### Flow cytometry

For HIV-1 latency-reversal assays, lentivector-transduced J-Lat cells were seeded in 48-well plates at a density of 5 × 10^5^ cells/well and treated with the indicated chemicals for 48 h. Then, the cells were collected, washed, and fixed for flow cytometry. For the cell viability assay, the cells were stained with eBioscience fixable viability dye (FVD) eFluor 780 (Invitrogen) at 4°C for an additional 30 min in the dark. The cells were subsequently analyzed via flow cytometry. A BD FACSCanto II flow cytometer (BD) was used to examine the percentage of GFP-positive cells and cell viability. Analysis of the acquired data was performed with FlowJo v10.

### Immunofluorescence imaging and bimolecular fluorescence complementation (BiFC) assay

The cells were fixed in 4% paraformaldehyde for 15 min and then permeabilized with 0.1% Triton X-100 for 5 min. After sufficient washes, the cells were blocked with 3% BSA for 30 minutes at room temperature and then incubated overnight with the indicated primary antibodies at 4°C. This was followed by incubation with Alexa Fluor-conjugated donkey anti-mouse or anti-rabbit secondary antibodies in the dark. Finally, the cells were incubated with Antifade Mounting Medium (containing DAPI, Beyotime). The samples were viewed via a FluoView FV3000 confocal microscope (Olympus).

The BiFC assay was performed as described previously [53]. Briefly, cells were seeded in a 35-mm glass bottom dish and transfected with appropriate BiFC constructs (0.5 μg each). At 24 h posttransfection, the cells were precultured at 4°C for 10 min and then stained with the appropriate antibodies as described above. These target fusion proteins were visualized via confocal microscopy. bJun-VN155 (I152L)/bFos-VC155 and bJun-VN155 (I152L)/bFosΔZIP-VC155 were chosen as controls.

### Ubiquitination assay

HEK293T cells were transiently co-transfected with His-tagged bait protein and YAP-Flag along with HA-tagged ubiquitin or not for 30 h. Then, the cells were incubated with 2.5 μM MG-132 for an additional 16 h. The cells were harvested and lysed in M2 buffer. The cell extracts were subsequently added to SDS to a final concentration of 1%, boiled for 5 min at 105°C, and diluted to 0.1% SDS with M2 buffer before immunoprecipitation. For the RNAi-ubiquitination experiments, the cells were first transfected with siRNA for 24 h before DNA transfection.

### Mass spectrometric (MS) analysis

Flag-tagged YAP and YAP mutants were transfected into HEK293T cells. Then, the Flag-tagged protein was extracted with anti-FLAG nanobody agarose beads (AlpalifeBio) via immunoprecipitation and separated via SDS-PAGE. The gel was digested with trypsin via an Easy-nLC 1200 (Thermo Fisher Scientific). Briefly, Protein samples (100 μg, amount adjustable per project) were digested with trypsin (1:20 enzyme-to-protein ratio, w/w) in 1.5 mL tubes after optional dilution with 0.5 M TEAB to ensure final concentrations <2 M urea and <0.1% SDS. Following 4 h incubation at 37°C, peptides were desalted and lyophilized. Dried peptides were reconstituted in mobile phase A (2% ACN, 0.1% FA), centrifuged (20,000 × g, 10 min), and the supernatant was subjected to nanoLC separation using an Easy-nLC 1200 system coupled to a self-packed C18 column (75 μm i.d. × 25 cm, 1.9 μm particles). Peptides were eluted at 200 nL/min with a gradient of mobile phase B (80% ACN, 0.1% FA): 5% B (0 ~ 3 min), 8 ~ 44% B (3 ~ 45 min), 44 ~ 60% B (45–50 min), 60 ~ 100% B (50 ~ 53 min), held at 80% B (53–60 min). Eluting peptides were ionized via nanoESI (2.1 kV) and analyzed on an Orbitrap Exploris 480 mass spectrometer operating in DDA mode. Full MS scans (350–1600 m/z) were acquired at 60,000 resolution; the top 2 precursors (charge states 2+ to 7 + , intensity >50,000) per cycle were selected for HCD-MS/MS (100 m/z start, 15,000 resolution, AGC target 1E5) with a 30 s dynamic exclusion. Finally, proteins were identified via MS by searching for matches in the UniProt protein database.

### GEO database analyses

The gene expression data for HIV-1 were downloaded from the GEO website, a public repository containing various forms of genetic information. The GSE140713 mRNA microarray, which included 50 AIDS patients and 7 uninfected healthy controls, was extensively investigated. The expression of YAP mRNA was obtained from the GEO2R database [54].

### Statistical analyses

The experimental data are presented as the means ± standard deviations (SDs) from at least three independent experiments. Statistical analyses were performed by using Prism 7.0 software (GraphPad). Comparisons between groups were performed via one-way analysis of variance (ANOVA) followed by Dunnett’s multiple-comparison post hoc test unless otherwise stated. A *p* value of <0.05 was used to indicate significance: *p < 0.05; **p < 0.01; ***p < 0.001.

## Supporting information

S1 Fig Analysis of YAP gene expression in AIDS patients stratified by viral load. YAP mRNA levels were compared between low viral load (LVL) and high viral load (HVL) groups of AIDS patients using the GEO2R online tool in the GSE140713 database. One-way ANOVA is conducted to detect significant differences.
(TIF)

S2 Fig Flow cytometric analysis of YAP knockdown in lentivirus-transduced J-Lat A2 cells. J-Lat A2 cell lines were infected with the pLKO.1-shYAP-mCherry lentivirus, and the YAP expression level was determined by western blotting. B) The cells were stimulated with or without LRAs for 48 h, and the GFP^+^ cells were determined by flow cytometry. Data are presented as mean ± SD. Two-way ANOVA is conducted to detect significant differences (*P < 0.05, ***P < 0.001).
(TIF)

S3 Fig Transcriptional kinetics upon YAP perturbation and rescue. TZM-bl cells with YAP knockdown (via siRNA) were transfected with a YAP expression plasmid, stimulated with TNFα, and subjected to luciferase assay to monitor HIV-1 LTR-driven transcriptional activity. Data are presented as mean ± SD. Two-way ANOVA is conducted to detect significant differences (***P < 0.001).
(TIF)

S4 Fig The association of YAP with the HIV promoter. CUT&Tag-qPCR analysis of the HEK293T cells cotransfected with pGL3–5’LTR-luc and YAP-Flag (OE-YAP) or empty vector (EV).
(TIF)

S5 Fig Confirmation of the binding mechanism between YAP and Tat. A) Co-IP analysis of the association of YAP and Tat in HEK293T cells transfected with the indicated exogenous gene expression vectors, with/without RNase A, by using a primary antibody. B) Co-IP analysis of the HEK293T cells cotransfected with YAP-Flag and Tat-His. C) Co-IP analysis of the HEK293T cells cotransfected with YAP-Flag and Tat-His or Tat^C22G^-His. D) Co-IP analysis of the HEK293T cells cotransfected with Tat-His, YAP-Flag and YAP truncation mutants.
(TIF)

S6 Fig Ubiquitination assay of His-tagged wild-type Tat or individual Lys-to-Arg mutants in HEK-293T cells cotransfected with YAP-Flag. HEK293T cells were co-transfected with plasmids encoding YAP-Flag and either His-tagged wild-type Tat or its individual Lys-to-Arg mutants. At 24–48 hours post-transfection, cells were treated with the proteasome inhibitor MG-132 before harvesting. Ubiquitination of Tat proteins was assessed by immunoprecipitation followed by immunoblotting with an anti-ubiquitin antibody.
(TIF)

S7 Fig Tat^K28,29,41R^ mutant restored the transcriptional activity lost by Tat^K41R^ mutant. CHX assay of His-tagged wild-type Tat and Lys-to-Arg mutants (K41R and K28,29,41R) in HEK293T cells. B) HIV-1 LTR-driven luciferase assay of TZM-bl cells transfected with His-tagged Tat^WT^, Tat^K29R^, Tat^K41R^, Tat^ΔK^, Tat^K28,29,41R^, Tat^K12,29,71R^, and vector for 48 h. C) HIV-1 LTR-driven luciferase assay of TZM-bl cells transfected with the indicated dose of His-tagged Tat^WT^, Tat^K28,29,41R^, Tat^K12,29,71R^, or vector before HIV-1_IIIB_ infection. Data are presented as mean ± SD. To detect significant differences, one-way ANOVA was conducted in panel B), and two-way ANOVA was conducted in panel C) (***P < 0.001).
(TIF)

S8 Fig The effect of E3s on the expression level and transcriptional activity of Tat. A) Venn diagram showing the overlap of the proteins among datasets obtained from IP-MS and the Public Library of Science. B) Immunoblot analysis of lysates of HEK293T cells co-transfected with Flag-tagged KCTD20, KLHL8, ZFPL1, and MID2 or vector and Tat-His for 48 h before harvest (bottom), and the protein expression was confirmed by Flag-trap co-immunoprecipitation assay (top). C) HIV-1 LTR-driven luciferase assay of TZM-bl cells transfected with KCTD20, KLHL8, ZFPL1, and MID2 or vector with/without Tat-His for 48 h.
(TIF)

S9 Fig Map of the domains of UHRF1 responsible for YAP binding. Flag-trap coimmunoprecipitation of HEK293T cells transfected with YAP-HA and indicated exogenous UHRF1 expression vector.
(TIF)

S1 Table RT-qPCR Primers for CUT&Tag-qPCR. (DOCX)
