# Supplementary figures and images for: YAP Inhibits HIV-1 transcription and promotes HIV-1 latency by regulating E3 ubiquitin ligase UHRF1 mediated tat degradation
Source: PLoS Pathog. 2026 Jan 30;22(1):e1013906. doi: 10.1371/journal.ppat.1013906 (PMC12857974; doi:10.1371/journal.ppat.1013906)

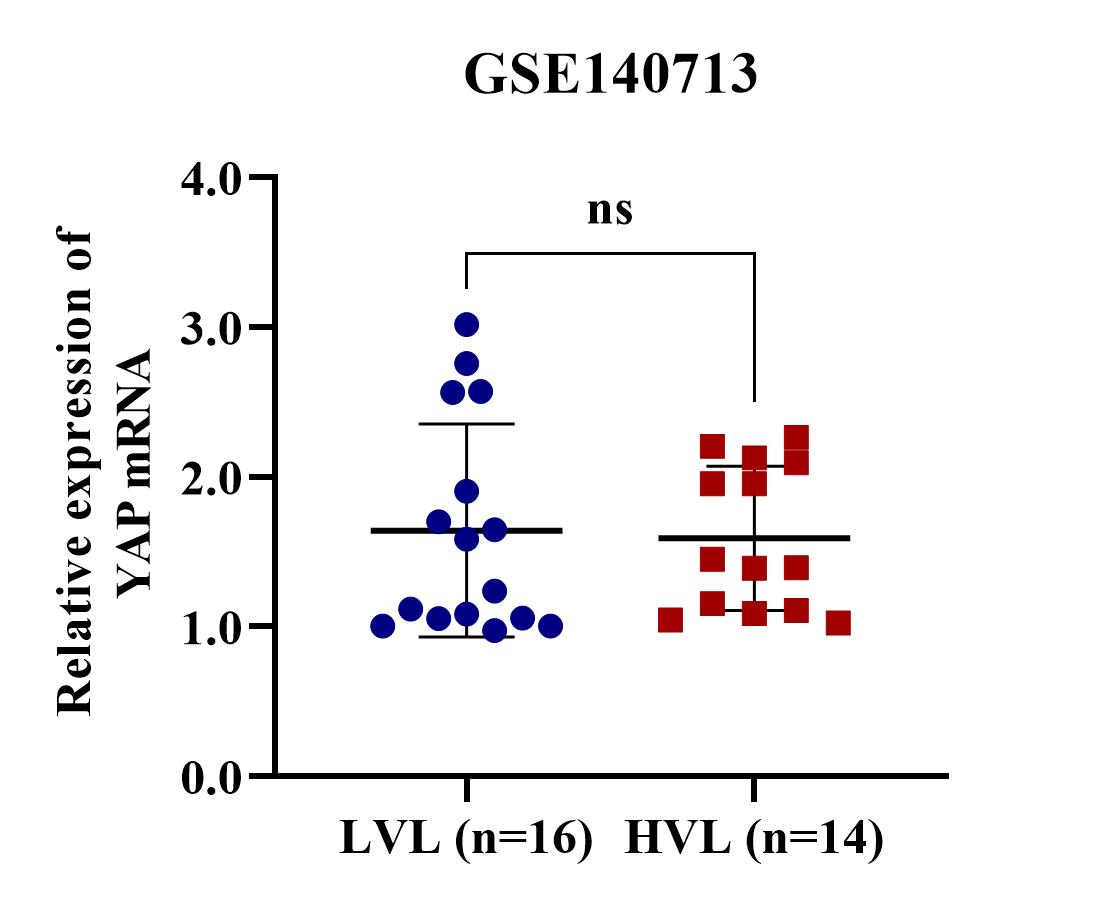

Supplement: S1 Fig — YAP mRNA levels were compared between low viral load (LVL) and high viral load (HVL) groups of AIDS patients using the GEO2R online tool in the GSE140713 database. One-way ANOVA is conducted to detect significant differences. (TIF) [file ppat.1013906.s001.tif]

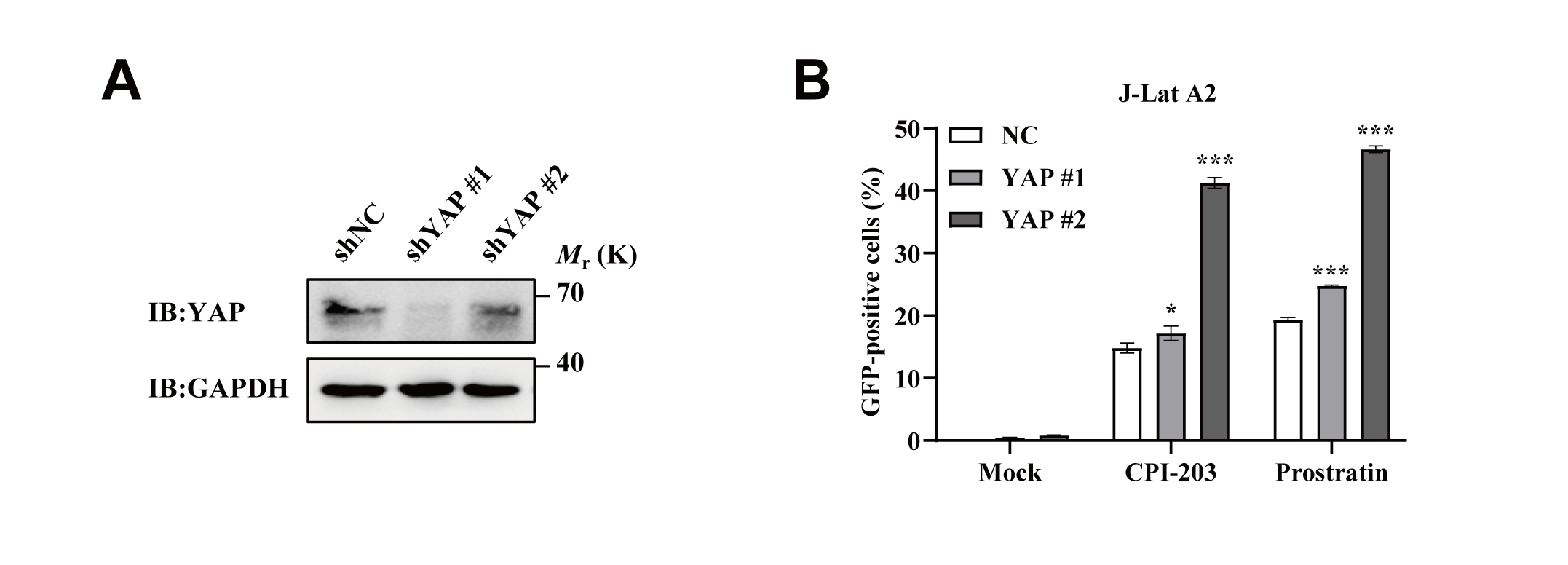

Supplement: S2 Fig — J-Lat A2 cell lines were infected with the pLKO.1-shYAP-mCherry lentivirus, and the YAP expression level was determined by western blotting. B) The cells were stimulated with or without LRAs for 48 h, and the GFP+ cells were determined by flow cytometry. Data are presented as mean ± SD. Two-way ANOVA is conducted to detect significant differences (*P < 0.05, ***P < 0.001). (TIF) [file ppat.1013906.s002.tif]

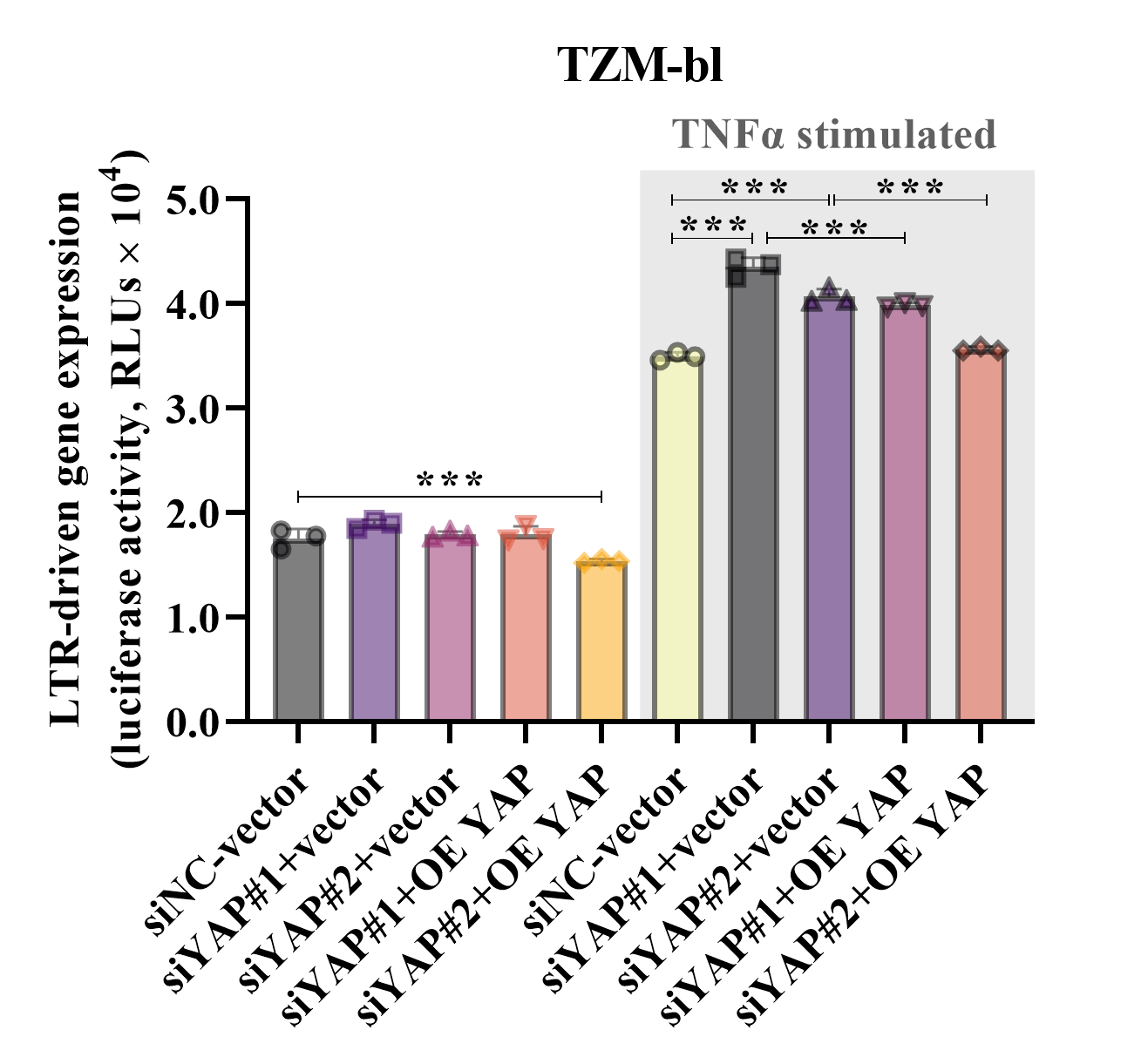

Supplement: S3 Fig — TZM-bl cells with YAP knockdown (via siRNA) were transfected with a YAP expression plasmid, stimulated with TNFα, and subjected to luciferase assay to monitor HIV-1 LTR-driven transcriptional activity. Data are presented as mean ± SD. Two-way ANOVA is conducted to detect significant differences (***P < 0.001). (TIF) [file ppat.1013906.s003.tif]

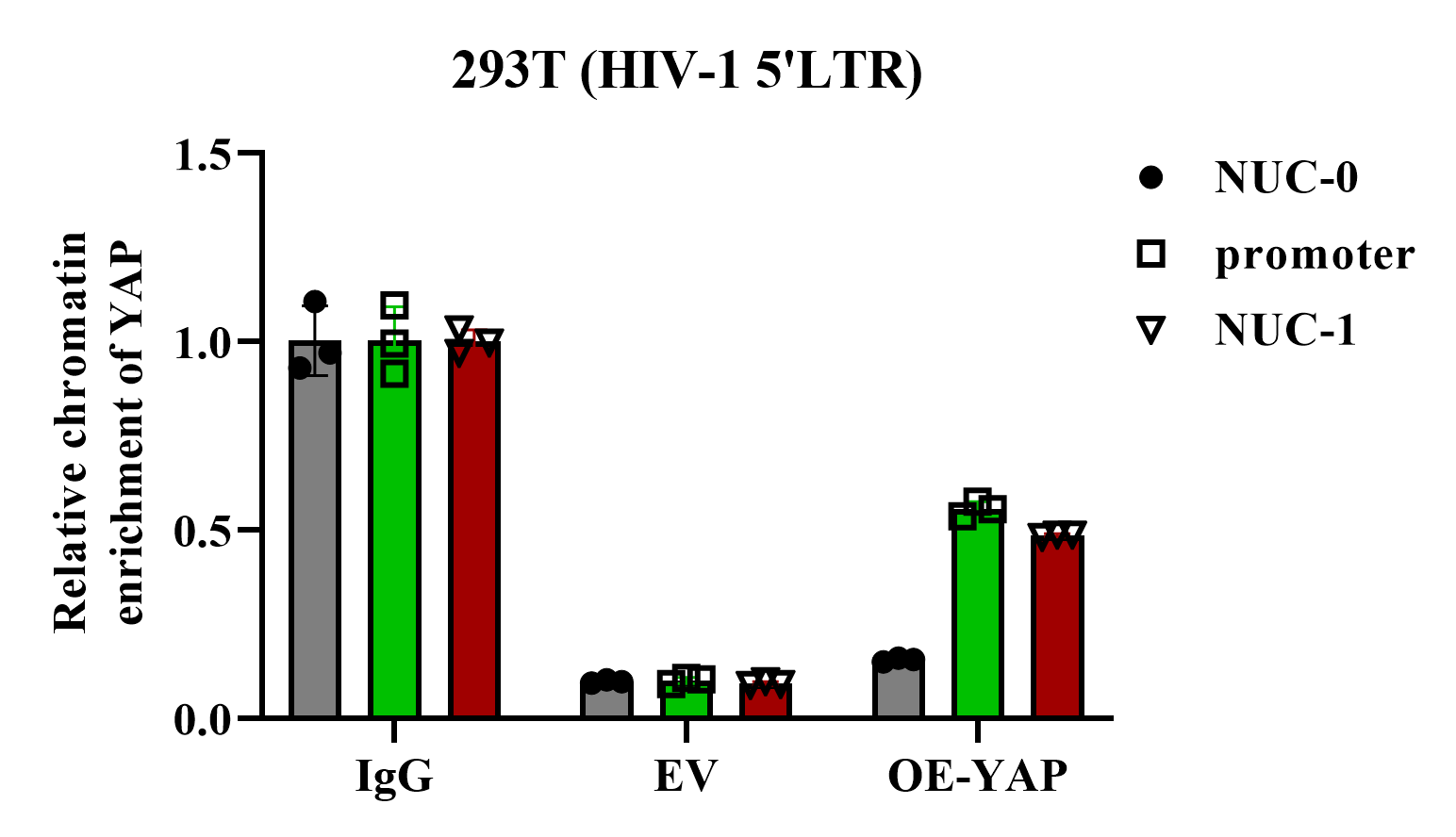

Supplement: S4 Fig — CUT&Tag-qPCR analysis of the HEK293T cells cotransfected with pGL3–5’LTR-luc and YAP-Flag (OE-YAP) or empty vector (EV). (TIF) [file ppat.1013906.s004.tif]

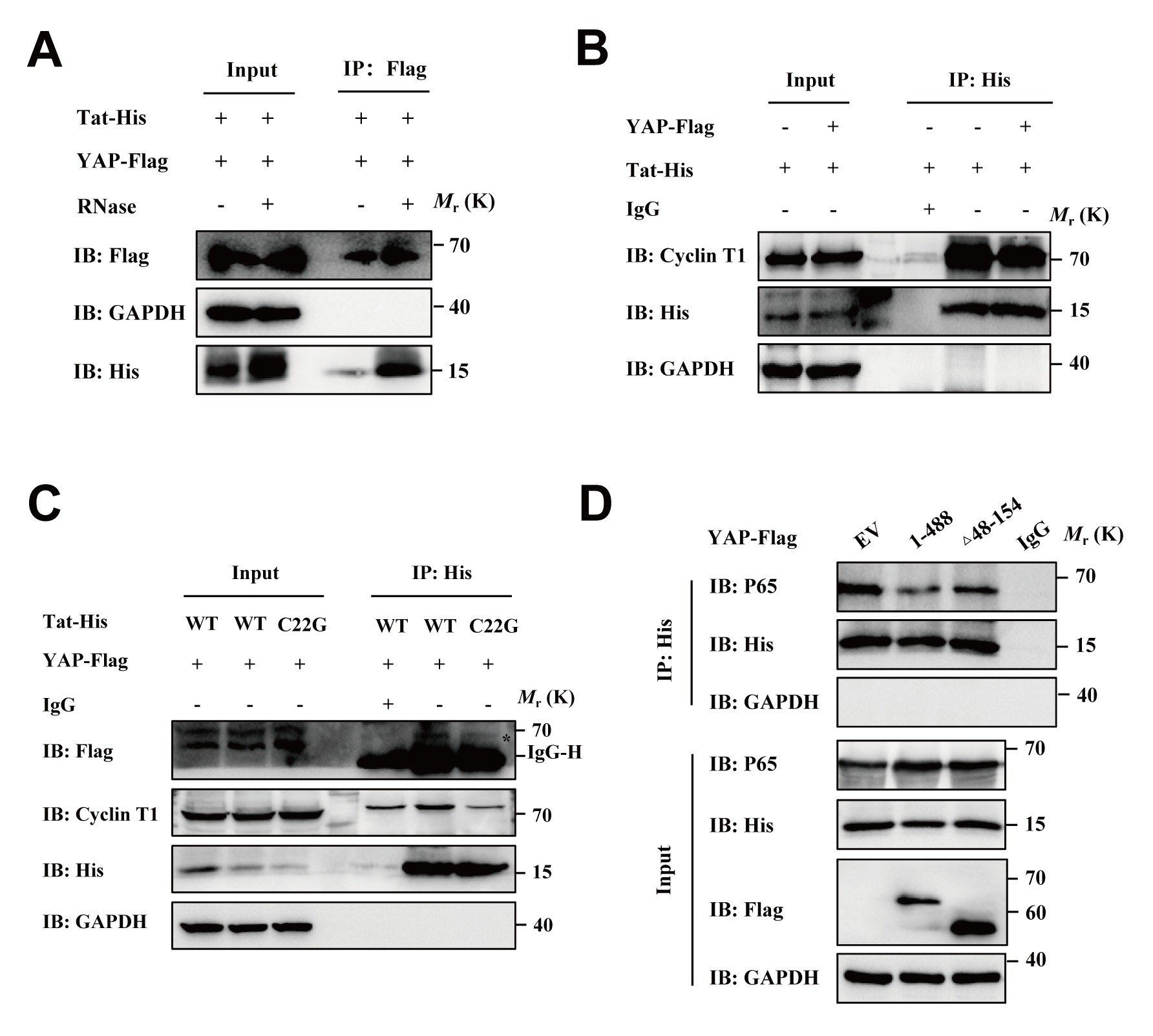

Supplement: S5 Fig — A) Co-IP analysis of the association of YAP and Tat in HEK293T cells transfected with the indicated exogenous gene expression vectors, with/without RNase A, by using a primary antibody. B) Co-IP analysis of the HEK293T cells cotransfected with YAP-Flag and Tat-His. C) Co-IP analysis of the HEK293T cells cotransfected with YAP-Flag and Tat-His or TatC22G-His. D) Co-IP analysis of the HEK293T cells cotransfected with Tat-His, YAP-Flag and YAP truncation mutants. (TIF) [file ppat.1013906.s005.tif]

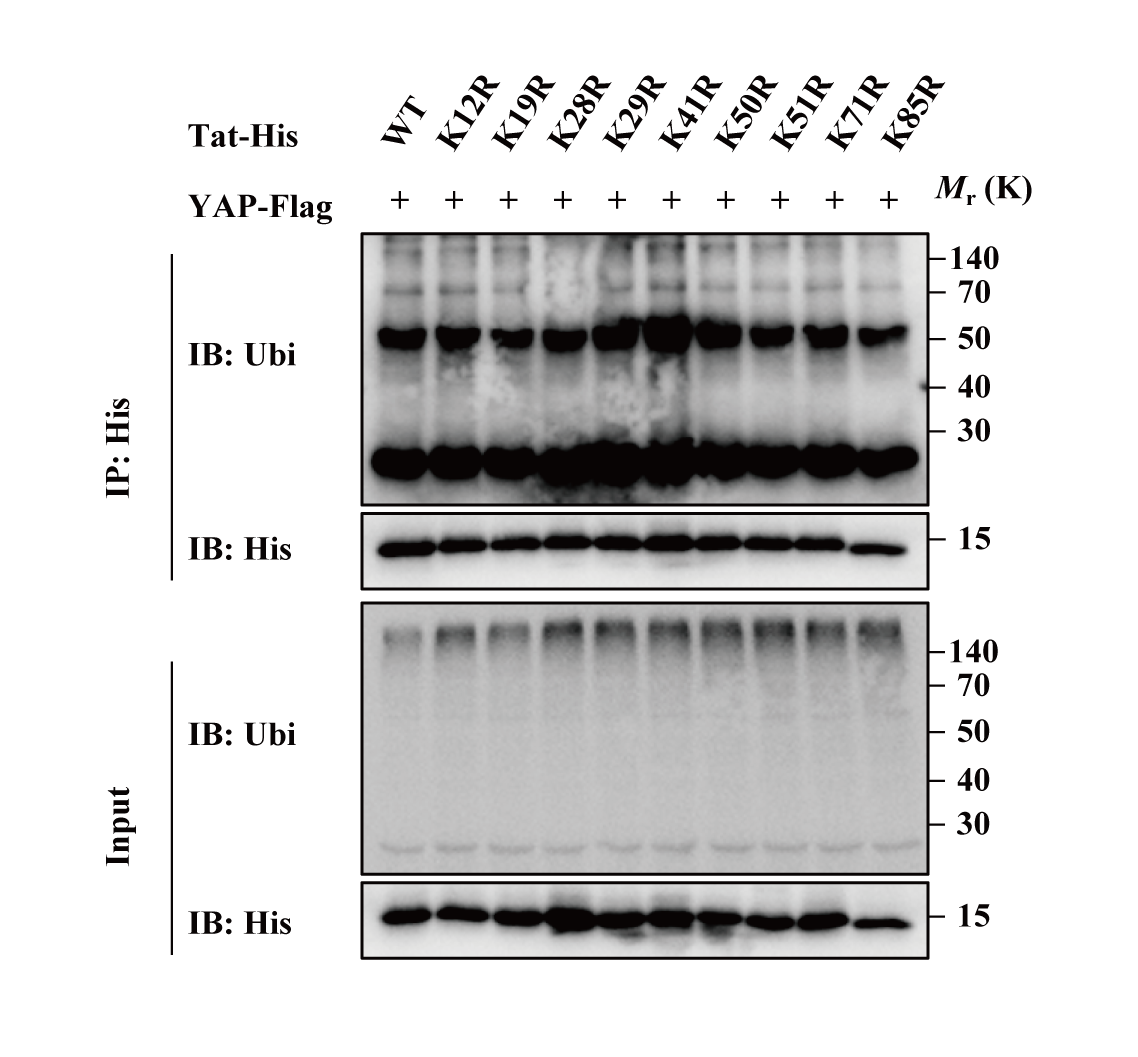

Supplement: S6 Fig — HEK293T cells were co-transfected with plasmids encoding YAP-Flag and either His-tagged wild-type Tat or its individual Lys-to-Arg mutants. At 24–48 hours post-transfection, cells were treated with the proteasome inhibitor MG-132 before harvesting. Ubiquitination of Tat proteins was assessed by immunoprecipitation followed by immunoblotting with an anti-ubiquitin antibody. (TIF) [file ppat.1013906.s006.tif]

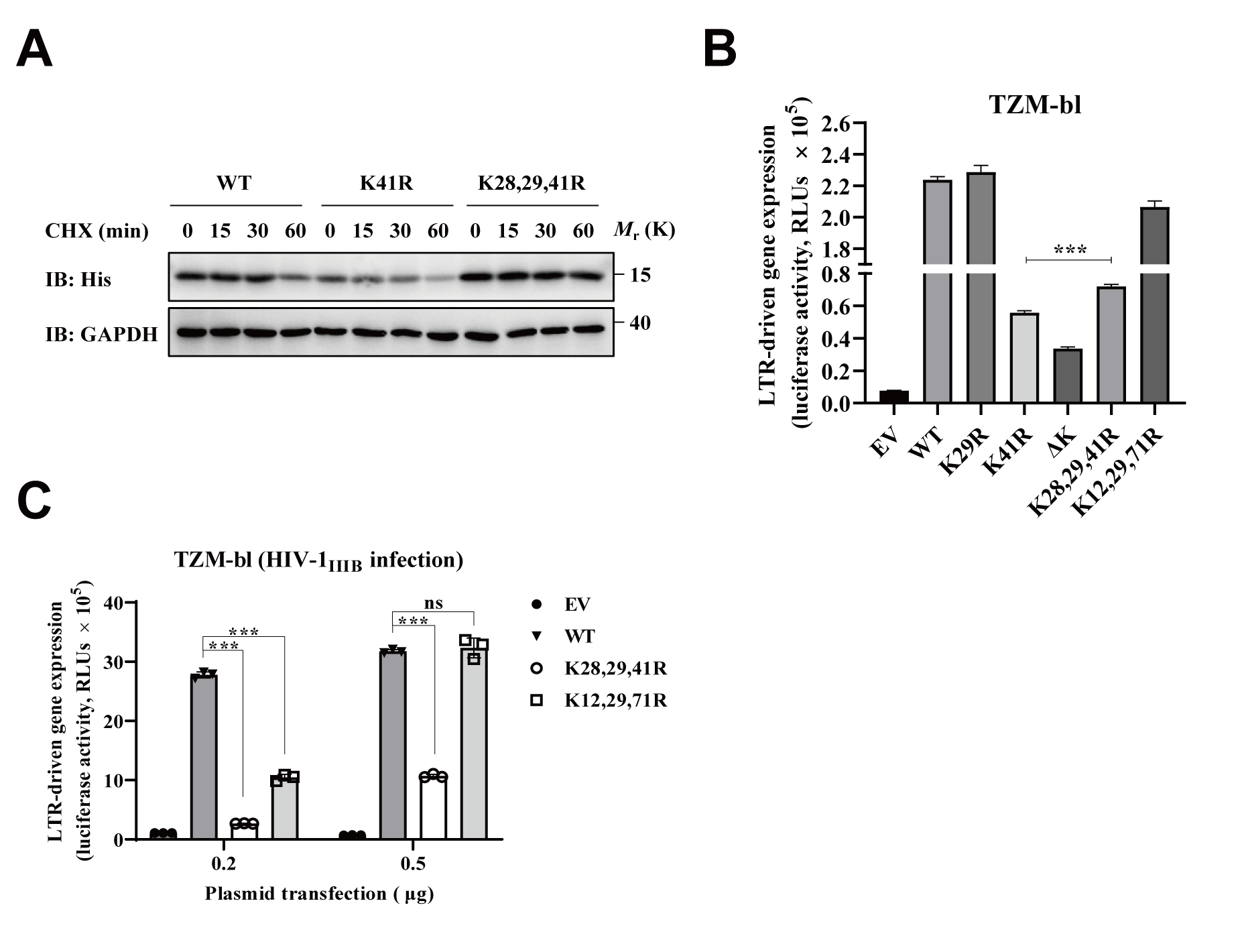

Supplement: S7 Fig — CHX assay of His-tagged wild-type Tat and Lys-to-Arg mutants (K41R and K28,29,41R) in HEK293T cells. B) HIV-1 LTR-driven luciferase assay of TZM-bl cells transfected with His-tagged TatWT, TatK29R, TatK41R, TatΔK, TatK28,29,41R, TatK12,29,71R, and vector for 48 h. C) HIV-1 LTR-driven luciferase assay of TZM-bl cells transfected with the indicated dose of His-tagged TatWT, TatK28,29,41R, TatK12,29,71R, or vector before HIV-1IIIB infection. Data are presented as mean ± SD. To detect significant differences, one-way ANOVA was conducted in panel B), and two-way ANOVA was conducted in panel C) (***P < 0.001). (TIF) [file ppat.1013906.s007.tif]

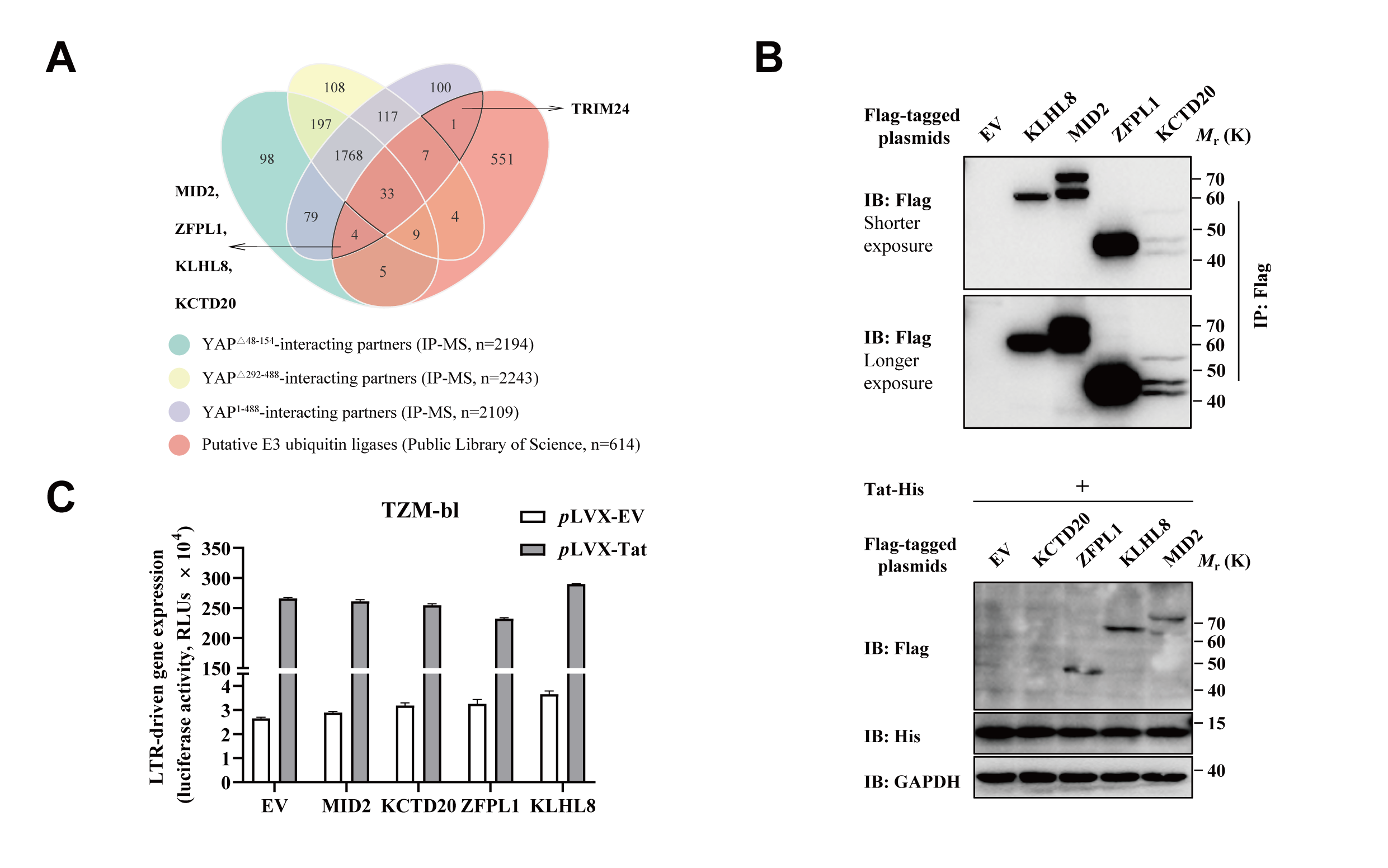

Supplement: S8 Fig — A) Venn diagram showing the overlap of the proteins among datasets obtained from IP-MS and the Public Library of Science. B) Immunoblot analysis of lysates of HEK293T cells co-transfected with Flag-tagged KCTD20, KLHL8, ZFPL1, and MID2 or vector and Tat-His for 48 h before harvest (bottom), and the protein expression was confirmed by Flag-trap co-immunoprecipitation assay (top). C) HIV-1 LTR-driven luciferase assay of TZM-bl cells transfected with KCTD20, KLHL8, ZFPL1, and MID2 or vector with/without Tat-His for 48 h. (TIF) [file ppat.1013906.s008.tif]

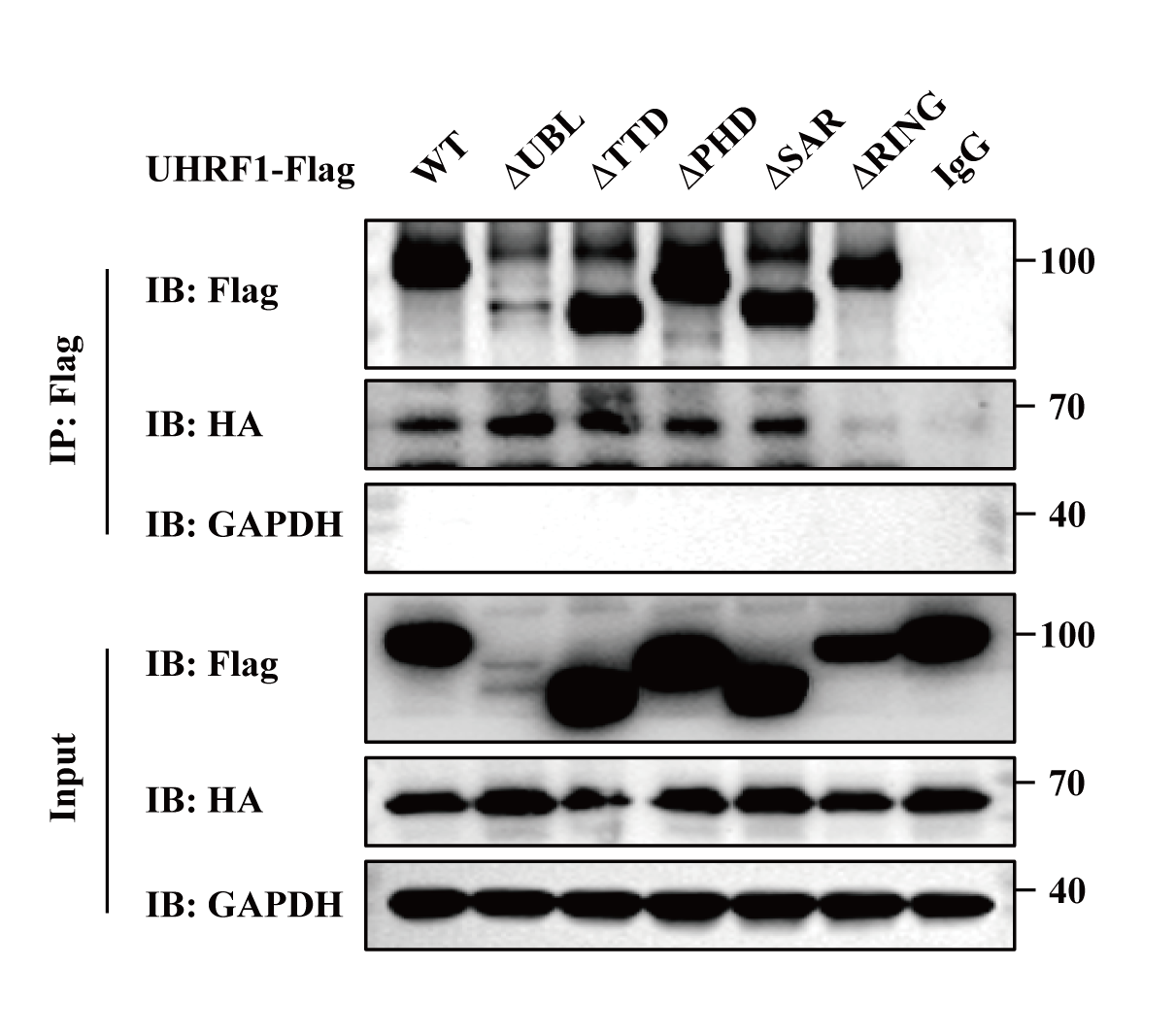

Supplement: S9 Fig — Flag-trap coimmunoprecipitation of HEK293T cells transfected with YAP-HA and indicated exogenous UHRF1 expression vector. (TIF) [file ppat.1013906.s009.tif]
