# Supplementary material for: YAP Inhibits HIV-1 transcription and promotes HIV-1 latency by regulating E3 ubiquitin ligase UHRF1 mediated tat degradation
Source: PLoS Pathog. 2026 Jan 30;22(1):e1013906. doi: 10.1371/journal.ppat.1013906 (PMC12857974; doi:10.1371/journal.ppat.1013906)
Supplement: S1 Table — (DOCX) [file ppat.1013906.s010.docx]

**S1 Table.** RT-qPCR Primers for CUT&Tag-qPCR.

| Primer name |  | Primer Sequence (5'-3') |
| --- | --- | --- |
| DNA Spike in | forward | GCCTTCTTCCCATTTCTGATCC |
|  | reverse | CACGAATCAGCGGTAAAGGT |
| Nuc0 | forward | TGGATCTACCACACACAAGG |
|  | reverse | GTACTAACTTGAAGCACCATCC |
| Nuc1 | forward | GCTTTTTGCCTGTACTGGGTCTCTC |
|  | reverse | CCACACTGACTAAAAGGGTCTGAGG |
| Promotor | forward | AGCTTGCTACAAGGGACTTTCC |
|  | reverse | ACCCAGTACAGGCAAAAAGCAG |
